# Successful Proof-of-Concept for Topical Delivery of Novel Peptide ALM201 with Potential Usefulness for Treating Neovascular Eye Disorders

**DOI:** 10.1016/j.xops.2022.100150

**Published:** 2022-04-04

**Authors:** Gideon Obasanmi, M. Andrew Nesbit, Diego Cobice, Logan Mackay, Stuart McGimpsey, Mark Wappett, Aaron N. Cranston, Tara C.B. Moore

**Affiliations:** 1 Biomedical Sciences Research Institute, School of Biomedical Sciences, Ulster University, Coleraine, United Kingdom; 2 Scottish Instrumentation and Research Centre for Advanced Mass Spectrometry (SIRCAMS), School of Chemistry, University of Edinburgh, Edinburgh, United Kingdom; 3 Mater Infirmorum Hospital (Belfast Health and Social Care Trust), Belfast, United Kingdom; 4 Centre for Precision Therapeutics, Almac Discovery Ltd., Belfast, United Kingdom

**Keywords:** Age-related macular degeneration, Angiogenesis, Choroidal neovascularization, Corneal vascularization, Ocular neovascularization, AMD, age-related macular degeneration, CNV, choroidal neovascularization, CV, corneal vascularization, HRA, Heidelberg retinal angiograph, nAMD, neovascular age-related macular degeneration, PBS, phosphate-buffered saline, ROI, region of interest, VEGF, vascular endothelial growth factor

## Abstract

**Purpose:**

To evaluate the therapeutic benefit of a novel peptide, ALM201, in ocular pathologic vascularization.

**Design:**

Experimental study in mouse, rat, and rabbit animal models.

**Participants:**

Ten-week-old Lister Hooded male rats, 8-week-old Brown Norway male rats, 9-day-old C57BL/6J mice, and 12-month-old New Zealand male rabbits.

**Methods:**

Corneal vascularization was scored for vessel density and vessel distance to suture in a rat corneal suture model. Ocular penetration and biodistribution were evaluated by matrix-assisted laser desorption/ionization mass spectrometry imaging after topical ALM201 application to rabbit eyes. A mouse choroidal sprouting assay, with aflibercept as positive control, was used to evaluate choroidal neovascularization (CNV) in the posterior segment tissue. Efficacy of topical ALM201 was assessed using a rat laser CNV model of neovascular age-related macular degeneration.

**Main Outcome Measures:**

Clinical scoring and histologic analysis of vascularized corneas, sprouting area, lesion size, and vessel leakiness in posterior segments.

**Results:**

Assessment of ALM201 treatment in the rat corneal suture model showed a significant decrease in vessel density (*P* = 0.0065) and vessel distance to suture (*P* = 0.021) compared with vehicle control (phosphate-buffered saline [PBS]). Infiltration of inflammatory cells into the corneal stroma also was reduced significantly compared with PBS (724.5 ± 122 cells/mm^2^ vs. 1837 ± 195.9 cells/mm^2^, respectively; *P* = 0.0029). Biodistribution in rabbit eyes confirmed ALM201 bioavailability in anterior and posterior ocular segments 1 hour after topical instillation. ALM201 treatment significantly suppressed choroid vessel sprouting when compared with PBS treatment (44.5 ± 14.31 pixels vs. 120.9 ± 33.37 pixels, respectively; *P* = 0.04) and was not inferior to aflibercept (65.63 ± 11.86 pixels; *P* = 0.7459). Furthermore, topical ALM201 significantly improved vessel leakiness (leakage scores: 2.1 ± 0.7 vs. 2.9 ± 0.1; *P* = 0.0274) and lesion size (144,729 ± 33,239 μm^3^ vs. 187,923 ± 28,575 μm^3^; *P* = 0.03) in the rat laser CNV model when compared with topical PBS vehicle.

**Conclusions:**

ALM201 is a promising novel molecule with anti-inflammatory and antivascularization activity and is a strong candidate to meet the clinical need of a new, topically delivered therapeutic agent for treating inflammation and pathologic vascularization in the anterior and posterior segments of the eye.

Angiogenesis is the process of growth of new blood vessels from preexisting ones.^1^^,^^2^ It is critical for normal physiologic bodily processes, such as reproduction, embryonic development, tissue growth, and wound healing^1^, ^2^, ^3^; however, dysregulated angiogenesis is implicated in various diseases, including cancer, rheumatoid arthritis, and, notably, vision-threatening microvascular diseases, including proliferative diabetic retinopathy, corneal vascularization (CV), neovascular age-related macular degeneration (nAMD), and retinopathy of prematurity.^2^, ^3^, ^4^ A number of these ocular neovascular conditions also are associated with an underlying inflammatory response.^5^^,^^6^ Of the leading global causes of blindness, age-related macular degeneration (AMD) and corneal opacities rank third and fourth, respectively.^7^ Both conditions significantly impact patient quality of life and present a considerable burden on health systems.

Within AMD, although the dry (nonexudative) form is more common, with a gradual loss of vision, the wet form (also called exudative AMD or nAMD) that is responsible for > 90% of AMD-related vision loss^8^ is characterized by a more rapid loss of central vision resulting from anomalous choroidal neovascularization (CNV) and sudden subretinal hemorrhage, which leaks intraretinal fluid into the macula, interfering with normal vision.^5^^,^^8^ If left untreated, this leads to fibrosis, scarring, and loss of vision. In the clinic, anti–vascular endothelial growth factor (anti-VEGF) therapies have become a mainstay treatment for many angiogenesis-related ocular conditions, including nAMD. Approved anti-VEGF biologicals include aflibercept (Eylea) and ranibizumab (Lucentis), whereas bevacizumab (Avastin) is used as an off-label treatment^9^, ^10^, ^11^; however, anti-VEGF agents are not effective treatments for all patients with nAMD because some do not respond to the treatment, whereas resistance develops in others over time. Long-term anti-VEGF use is also under debate because multiple intravitreal injections of expensive biological drugs are needed to slow disease progression^11^ and do not effectively treat fibrosis and may even exacerbate it.

Similarly, at the front of the eye, where a transparent, nonvascularized cornea is critical for optimal vision, CV is characterized by proliferation of blood vessels from the capillaries and venules of the pericorneal plexus at the limbus into the normally avascular central cornea.^12^^,^^13^ Where CV, inflammation, or both has occurred, visual acuity is reduced, and if left untreated, eventual blindness can ensue.^14^ Clinically, CV is managed mainly by topical administration of steroidal and nonsteroidal anti-inflammatory drugs as first-line treatments^15^; however, steroids increase the risk of superinfection, glaucoma, cataracts, and herpes simplex recurrence, whereas nonsteroidal anti-inflammatory drug use often is ineffective and increases the risk of corneal ulceration and melting.^14^, ^15^, ^16^, ^17^, ^18^ Another prevailing treatment for vascularization-induced corneal opacity is corneal graft; however, CV significantly increases the risk of graft rejection, regardless of whether it is a penetrating or lamellar keratoplasty.^15^^,^^19^, ^20^, ^21^ Other treatments include the use of anti-VEGF drugs as antiangiogenics and fine-needle diathermy, but risks also accompany these treatments, including epithelial defects and stromal thinning for anti-VEGF treatments and intracorneal bleeding, corneal perforation, and temporary corneal opacification for fine-needle diathermy treatments.^14^^,^^15^

Given that current available therapies often have limited efficacy and associated deleterious side effects, alternative effective, safe, and noninvasive therapies are needed for sight-limiting ocular conditions in which inflammation and pathologic neovascularization are implicated. Among anti-inflammatory and antineovascularization treatment options with potential therapeutic usefulness in neovascular ocular conditions are the therapeutic peptides derived from the N-terminal region of the immunophilin family member, FK506-binding protein like, which possess, among other biological properties, novel antiangiogenic activity.^22^ FK506-binding protein like and its derivatized peptides have been observed to affect microtubule formation, endothelial cell migration, and angiogenesis in a range of in vitro and in vivo assays via components of a CD44-dependent pathway.^22^, ^23^, ^24^ The smallest of these FK506-binding protein like−derived synthetic peptides, ALM201, is a 23-amino acid molecule that previously was shown to be antiangiogenic in preclinical tumor studies. ALM201 was progressed successfully through good laboratory practice toxicology studies and taken into phase Ia clinical trials, where it was shown to be safe when dosed systemically to patients with advanced solid tumors (European Union Drug Regulating Authorities Clinical Trials identifier: 2014-001175-31).^25^^,^^26^

Given these results, we explored the potential therapeutic benefits of using ALM201 as a topical agent to treat ophthalmic disorders where inflammation and neovascularization were the underlying pathologic conditions. The effectiveness and local tolerability of topical ALM201 were assessed in anterior and posterior segments of the eye using several industry-standard preclinical models. Our data strongly support a potential clinical usefulness for this novel therapeutic peptide in ocular conditions with underlying angiogenic and inflammatory pathogenesis.^27^^,^^28^

## Methods

### Animals

Male Lister hooded rats, C57BL/6J mice, and New Zealand white rabbits were purchased from Envigo. Brown Norway male rats were sourced from Charles River. Animals were acclimated for at least 7 days and housed in a pathogen-free setting with 12-hour light–dark cycles and controlled temperature (22 ± 1 °C) and humidity (55 ± 5%) ranges and access to food and water ad libitum. Animals were cared for by trained personnel, and procedures were conducted by trained, licensed scientists. All procedures were conducted in accordance with the United Kingdom Animals (Scientific Procedures) Act 1986 and approved by the Department of Health, Social Services, and Public Safety of Northern Ireland. For laser CNV experiments performed by Iris Pharma (La Gaude, France), all procedures and protocols were reviewed by an internal ethics committee (identifier: DAP53), and animals were treated according to Directive 2010/63/EU of the European Convention for the Protection of Vertebrate Animals Used for Experimental and Other Scientific Purposes. All experiments were conducted in compliance with the Association for Research in Vision and Ophthalmology Statement for the Use of Animals in Ophthalmology and Vision Research.

### Rat Corneal Suture Model

Under general anesthesia (ketamine and xylazine), local anesthetic (tetracaine hydrochloride) was applied topically to the eyes of 10-week-old Lister hooded rats. Corneal vascularization was induced by an intrastromal suture with a 10-0 Ethilon nylon suture (Ethicon) into the temporal cornea of each eye; the distance from the limbus of which was measured using a Vernier caliper set to 1.5 mm. Twenty-four hours later, sutured eyes were treated topically with 16 μl of ALM201 (100 nM) in phosphate-buffered saline (PBS), whereas the contralateral eye was treated with PBS alone. Topical treatment with ALM201 was continued once every 24 hours for 6 days. After 7 days, corneal images were acquired using a surgical microscope camera (Leica Microsystems), and the images were scored clinically for (1) vessel density (0 = no density, 1 = low density, 2 = medium density, and 3 = high density), (2) vessel distance to suture (0 = no reach, 1 = small reach, 2 = moderate reach, 3 = three-quarters distance reach, and 4 = reached sutures), and (3) inflammation (0 = none, 1 = mild, 2 = moderate, and 3 = severe; Supplemental Fig S1). Clinical scoring was carried out by 2 different investigators who were masked to the treatment groups. Thereafter, animals were euthanized by inhalation to a slowly rising concentration of CO_2_, and death was confirmed by a second approved humane method. Whole eyes were enucleated for further immunohistochemical analysis.

### OCT

After corneal sutures were placed, pupils of rats were dilated, and corneal structural changes induced by the sutures were assessed immediately using OCT (Phoenix Micron IV Imaging Microscope; Phoenix Research Labs) according to the manufacturer’s instructions. Slit-lamp, live photographs of the cornea were captured alongside corneal OCT scans.

### Histologic and Immunohistochemistry Examination

Eyes were fixed in 2% paraformaldehyde overnight before being processed and embedded in paraffin wax. Thereafter, tissue was cut into 5-μm thick sections using a microtome (Leica RM 2135) and left overnight at 37 °C. The sections then were deparaffinized in xylene (twice for 5 minutes) and rehydrated in a gradient of ethanol (100%, 95%, and 75%, all twice for 5 minutes) and stained with hematoxylin–eosin to identify infiltrating cells. Slides were scanned digitally at ×20 magnification using a Hamamatsu Nanozoomer (Hamamatsu Photonics) in brightfield mode (OracleBio; BioCity). Infiltrating cells were counted in the corneal stroma region of interest with NanoZoomer Digital Pathology. Version 2.0, viewer software (Hamamatsu Photonics) by a masked investigator to avoid bias. Counted cells were normalized to area (square millimeters).

### Choroidal Sprouting Assay

The detailed protocol of this assay is described elsewhere.^29^ Briefly, 9-day-old C57BL/6J mice were euthanized by a slowly rising concentration of CO_2_ inhalation, and death was confirmed by a second approved method. Eyes were enucleated, and the peripheral choroidoscleral complex was dissected, cut into 1-mm^2^ fragments, and grown on growth factor-reduced Matrigel (ThermoFisher) at 37 °C with 5% CO_2_ for 48 hours before treatments (aflibercept [0.5 mg/ml], ALM201 [100 nM], or PBS) were administered. Phase contrast photographs of individual explants were obtained every 48 hours, and the areas of sprouting were quantified with ImageJ software (National Institutes of Health).

### Mass Spectrometry Imaging

ALM201 (100 nM) or PBS (contralateral eye) was applied topically (30 μl) to the eyes of 12-month-old male New Zealand rabbits. One hour later, rabbits were euthanized by ketamine administration, and death was confirmed by a second approved method. Eyes were enucleated immediately and stored at –80 °C until tissue cryosectioning using a Leica CM 1850 cryostat (Leica Biosystem). Ten-micrometer cross-sections were collected from the center of the eye and placed on indium tin oxide−coated matrix-assisted laser desorption ionization imaging slides (Bruker Daltonics GmbH). Slides then were vacuum desiccated for 40 minutes and coated with α-cyano-4-hydroxycinnamic acid matrix-assisted laser desorption ionization matrix, 5 mg/ml in 80:20 (acetonitrile: water with 0.1% volume/volume of trifluoroacetic acid) using a modified 3-dimensional printer as described previously.^30^ Three-dimensional printer parameters were set as follows: pump flow, 0.1 ml/minute; nitrogen pressure, 2 bar; number of passes, 4; and distance from the nozzle to the target (z), 15 mm. Matrix density was 0.12 mg/cm^2^.

Mass spectrometry analysis was performed using a 12-Tesla SolariX matrix-assisted laser desorption ionization-Fourier transform ion cyclotron resonance mass spectrometer (Bruker Daltonics GmbH) using a Smart beam 2-kHz laser, with instrument control using SolariX control, version 1.5.0 (build 42.8). Ions were accumulated across 850 laser shots with a laser spot diameter of approximately 20 μm, and laser power was optimized for consistent ion production. Raster was set at 100 × 100 μm at 45-μm lateral resolution. Ions were detected using broadband (250–3000 Da) in positive ion mode with a 4 Mword (1 Mword is equal to 4 bytes) time-domain transient. Monoisotopic mass for ALM201 of interest was confirmed using Data Analysis 4.4, built 4.4.200 (Bruker Daltonics GmbH). The product ion was identified using an error tolerance of 10 ppm for the corresponding theoretical monoisotopic masses. Peak intensities of ALM201 were normalized using root mean square analysis using Fleximaging, version 4.1 (Bruker Daltonics GmbH).

### Rat Laser CNV Efficacy Study

Experimental work for the rat laser CNV study was performed at Iris Pharma. Brown Norway male rats were assessed daily and were approximately 8 weeks of age at the start of the experiment. Only healthy animals with no visible sign of ocular defect were assigned randomly to the study groups in 3 series. Choroidal neovascularization was induced by applying 170 mW of 532-nm laser light from a photocoagulator (6 75-μm spots for 0.1 second) in the right eyes on day 0. Ocular examinations were performed on both eyes using a slit lamp (anterior and posterior segments) at baseline and on days 3, 7, 13, and 20. Fluorescein angiography in the right eyes was evaluated on days 14 and 21 using the Heidelberg retinal angiograph (HRA; Heidelberg Engineering). Lesion size was determined at the end of the study (day 23) on choroid flat mounts labeled with isolectin B4.

### Laser Induction of Choroidal Neovascularized Lesions

Animals were anesthetized by an intramuscular injection of a Rompun (xylazine) and Imalgene (ketamine) mixture before lesion induction, HRA evaluations, and topical drug administration. Right-eye pupils underwent dilatation by instillation of 1 drop of 0.5% tropicamide before lesion induction and HRA examination; a topical anesthetic (diluted Cebesine) was applied before lesion induction and intravitreal dosing. Six neovascularized lesions were induced in the right eye by applying 170 mW of 532-nm laser light (Viridis laser; Quantel) on 75-μm areas around the optic nerve, between the main retinal vessel branches, for 0.1 second, through the slit lamp and a contact lens. Production of a bubble at the time of laser application confirmed the rupture of Bruch’s membrane. If a choroidal hemorrhage bigger than the diameter of the lesion was observed, then the lesion was excluded, and another lesion was induced.

### Treatment Doses and Schedules

Animals were anesthetized, and ALM201 (10 μM in PBS) or vehicle (PBS) was administered topically (20 μl) to right eyes (after lesion induction) twice daily (3.5-hour interval) on days 0 through 21. A dose of 10 μM was chosen for the rat laser CNV study. This dose was used to ensure sufficient delivery to the back of the eye and was based on a multiple of the doses used to achieve efficacy in the rat corneal suture wound model. This dose is multiple orders of magnitude lower than the amount used systemically in solid tumor studies with ALM201. Pilot experiments with topical ALM201 biodistribution studies in rat eyes showing optimal biodistribution in the eye also informed the dose used. Aflibercept (40 mg/ml) was injected intravitreally (5 μl = 0.2 mg) into the right eyes of a comparator group of animals on days 0, 3, 7, 10, 13, and 17 under anesthesia using an operating microscope and a 30-gauge needle mounted on a 100-μl Hamilton syringe. Body weights and clinical signs of all animals were recorded daily. Both eyes of each rat were examined using a slit lamp and were scored using the McDonald−Shadduck scale^31^^,^^32^; the fundus was observed when possible.

### Vessel Angiography and Scoring

Fluorescein angiography was performed on the lesions using an HRA under anesthesia. Ten percent sodium fluorescein (250 μl/100 g body weight) was injected subcutaneously, and fluorescence photographs were obtained 10 ± 2 minutes after dye injection. The leakage of fluorescein was evaluated in the angiograms by 2 examiners masked to the study groups and graded for fluorescein intensity as follows: 0 = no staining, 1 = slightly stained, 2 = moderately stained, and 3 = strongly stained. Where there was a discrepancy, the higher of the 2 scores was used. A notation of not determined (ND) was indicated if (1) an anomaly of the eye was observed and the angiogram was not evaluable; (2) a lesion was fused with another lesion, in which case both lesions were excluded; or (3) if > 3 lesions in an eye were excluded; then all the lesions in that eye were excluded.

### Choroidal Flat-Mount Staining and Lesion Quantitation

On day 23, animals were euthanized by an intraperitoneal injection of pentobarbital after sedation, and death was confirmed by a second humane method. Immediately after death, the right eye was collected and fixed in 4% paraformaldehyde for 1 hour at room temperature. After washing with PBS, the retina-choroid-sclera layers were dissected, and the retina was separated carefully. The remaining retinal pigmented epithelium choroidoscleral tissue was flat mounted and labeled with fluorescein isothiocyanate-isolectin B4 (Vector Laboratories). The volume of the neovascular lesions was quantified using stacked images acquired at a 488-nm excitation wavelength using an ApoTome fluorescence microscope (Zeiss) and was analyzed using AxioVision imaging software (Zeiss). Choroidal neovascularization area on each z-stack image was quantified using software that outlines the border of neovessels, and the volume was calculated with the sum of each area multiplied by the distance between each z-stack image. A lesion was excluded if (1) it was fused with another lesion or if it was larger than the visual field of the microscope (outlier); (2) an artifact (e.g., folds, fragments) covered the lesion; or (3) choroidal hemorrhage was present.

### Statistical Analysis

Summary statistics such as mean, median, and standard deviations were calculated. All tests were carried out using GraphPad Prism, version 6, software (GraphPad). Normality was tested in continuous variables with the D’Agostino−Pearson omnibus normality test. All data then were expressed as either mean and standard error of the mean or median with lower and upper quartiles plus minimum and maximum scores, as applicable. Comparisons between 2 groups of continuous variables were carried out with independent samples *t* test, whereas comparisons among > 2 groups of continuous variables were carried out with a 1-way analysis of variance and subsequent Tukey’s multiple comparisons post hoc test. Comparisons between 2 groups of ordinal variables were carried out with the Mann–Whitney *U* test. *P* values < 0.05 were considered statistically significant. According to the distribution of data for angiography scoring and neovascularization size in the rat laser CNV model, a Kruskall−Wallis test was performed on the median individual data to compare the different groups, or Dunn’s multiple comparisons test was performed. Additional statistical lesion-by-lesion analysis (using R, version 3.4; R Foundation for Statistical Computing) on angiography scoring and neovascularized lesion size also was performed using the Wilcoxon ranked-sum test. No normal distribution was assumed, and a nonparametric test was appropriate. The analysis was performed 2 × 2 and is represented by scatterplots. Boxplot upper and lower hinges correspond to the 25th and 75th percentiles with the midpoint corresponding to the median. Whiskers extend 1.5 × interquartile range of the hinge. Only *P* values corresponding to a comparison between topical vehicle versus topical ALM201 (10 μM) or versus intravitreal aflibercept (Eylea) were reported directly on the graphs.

## Results

### Topical Application of ALM201 Suppresses Corneal Neovascularization and Inflammation

**Figure 1:**
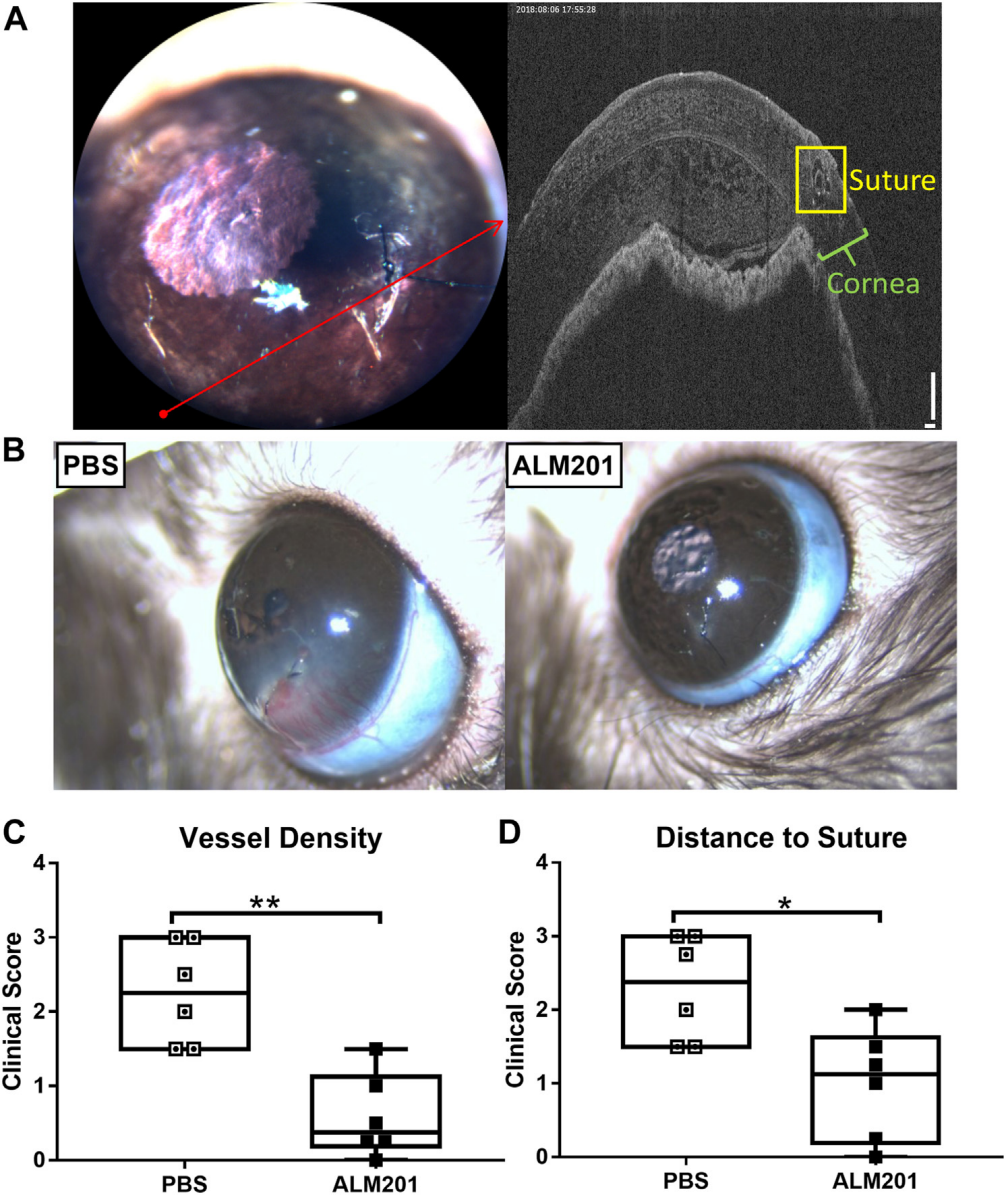
Effects of ALM201 on the hallmarks of neovascularization in a rat suture model of corneal vascularization. **A**, Representative slit-lamp biomicroscope photograph of the cornea (left) and corneal OCT scan (right) obtained after suture placement. Red arrow on corneal slit-lamp biomicroscope photograph indicates region of representative corneal OCT scan. Representative corneal OCT scan shows sutures placed in the corneal stroma without anterior chamber perforation. **B**, Representative images of sutured corneas after 6 days of treatment with vehicle (phosphate-buffered saline [PBS]; left) and ALM201 (right). **C**, **D**, Box-and-whisker plots showing clinical scoring of sutured corneas after 6 days of once-daily treatment with either vehicle (PBS) or ALM201 (16 μl, 100 nM): (**C**) vessel density and (**D**) distance to suture. The central bar indicates the median score, the ends of the boxes show the upper and lower quartiles, and the whiskers indicate the minimum and maximum scores (n = 6). ∗*P* < 0.05; ∗∗*P* < 0.01, Mann–Whitney *U* test.

Corneal neovascularization was induced by the placement of an intrastromal suture into the temporal cornea. Immediate OCT scans after suture placement showed that the sutures did not penetrate the anterior chamber (Fig 1A). Compared with vehicle (PBS), ALM201 markedly inhibited neovascularization with minimal vessel growth from the limbus toward the sutures, and no scarring, edema, whiteness, or other signs of inflammation were observed (Fig 1B). Clinical scores of corneal vascular response, vessel density (Fig 1C; *P* = 0.0065), and distance to suture (Fig 1D; *P* = 0.021) were reduced significantly in ALM201-treated sutured eyes compared with PBS-treated sutured eyes. The reduced vessel density and reduced vessel distance to suture after topical treatment with ALM201 improve both light transmission and visual acuity.

### Topical Application of ALM201 Suppresses Stromal Cellular Infiltration

**Figure 2:**
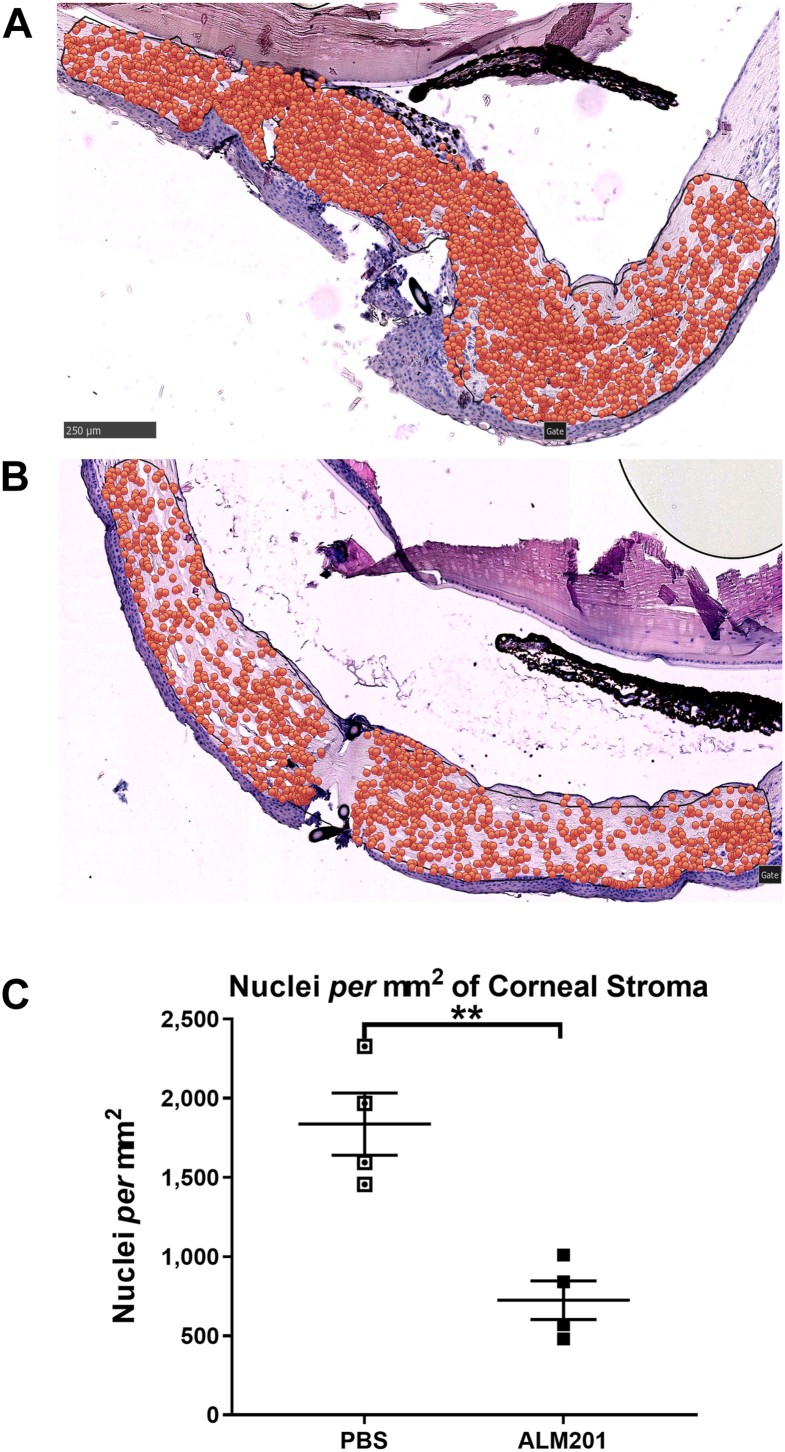
ALM201 reduces corneal stromal cellular infiltration in the rat suture model of corneal vascularization. **A**, **B**, Representative histologic images from sutured rat eyes treated once daily for 6 days (stain, hematoxylin–eosin, original magnification 20×). Superimposed orange dots represent cell nuclei. Scale bar, 250 μm. **A**, Vehicle (phosphate-buffered saline [PBS])-treated eyes showing marked cellular infiltration. **B**, ALM201-treated sutured eyes showing reduced cellular infiltration. **C**, Graph showing nuclei per square millimeter of corneal stroma in PBS- and ALM201-treated sutured eyes. Results are presented as mean ± standard error of the mean (n = 4). ∗∗*P* < 0.01, independent samples *t* test.

Cellular infiltration, a hallmark of vascularization-related inflammation observed in the eyes of PBS-treated rats in the suture model of CV (Fig 2A), was suppressed by topical ALM201 treatment (Fig 2B). Eyes treated with topical application of 100 nM ALM201 (16 μl) once daily for 6 days resulted in a significantly reduced number of nuclei per square millimeter of corneal stroma when compared with PBS treatment (724.5 ± 122 cells/mm^2^ vs. 1837 ± 195.9 cells/mm^2^, respectively; *P* = 0.0029; Fig 2C).

### Biodistribution of ALM201 after Topical Application to the Rabbit Eye

**Figure 3:**
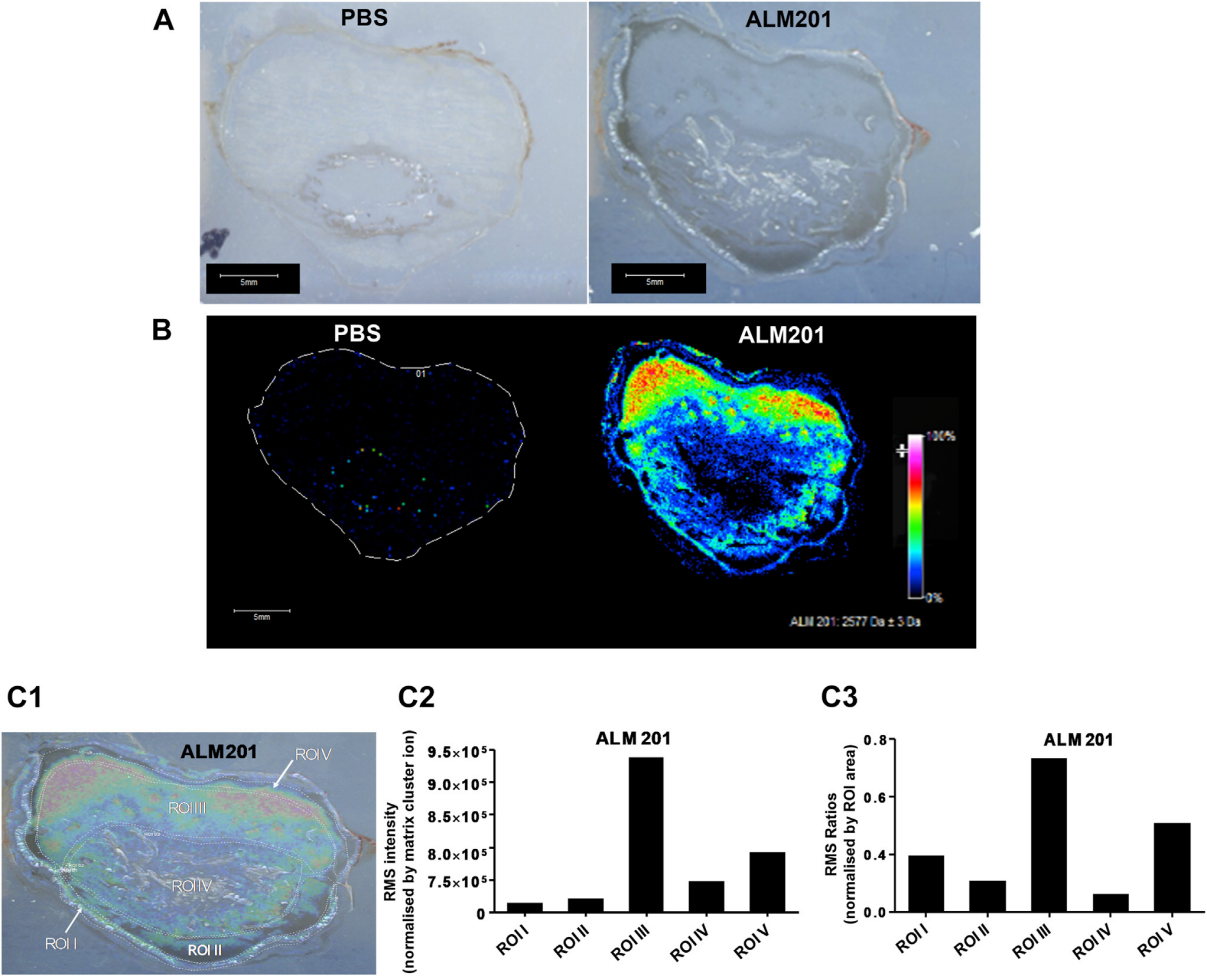
Ocular biodistribution of ALM201 full-length parent peptide 1 hour after a single topical dose (100 nM) detected by matrix-assisted laser desorption ionization (MALDI)-Fourier transform ion cyclotron resonance mass spectrometry (FTICR-MS) directly on representative rabbit ocular sections. **A**, Optical images of mass spectrometry imaging analyses of rabbit eye tissue sections treated with vehicle (phosphate-buffered saline [PBS]; left) or ALM201 (right). Scale bar, 5 mm. **B**, Heat map of ALM201 at mass-to-charge ratio (m/z) 2575 ± 2 Da in rabbit eye tissue sections collated by MALDI-FTICR-MS treated with PBS vehicle (left) or ALM201 (right). Scale bar, 5 mm. Signal intensity is depicted by color on the scale shown normalized by root mean square (RMS) analysis. Molecular regional distribution maps showed ALM201 (m/z 2575) in high abundance mainly distributed across aqueous and vitreous humor with mass accuracy of ± 10 ppm from their theoretical monoisotopic masses and signal-to-noise ratios of > 100. **C**, Regional intensity distribution of ALM201 in selected regions of interest (ROIs) after topical treatment: ROI I, cornea; ROI II, aqueous humor; ROI III, vitreous humor; ROI IV, lens; and ROI V, retina/choroid. **C1**, Heatmap-optimal image overlay of ALM201 at m/z 2575 ± 2 Da in selected ROIs. **C2**, Absolute RMS intensity of selected ROIs. **C3**, Regions of interest normalized by intensity of selected ROIs.

Initial rat eye studies demonstrated the ability of matrix-assisted laser desorption ionization mass spectrometry imaging to assess ALM201 distribution throughout the eye (unpublished observations). Histologic cross sections of rabbit eyes before matrix-assisted laser desorption ionization mass spectrometry imaging did not show any irregularities (Fig 3A). Compared with the PBS-treated eye, in which no signal was observed (Fig 3B), 1 hour after topical ALM201 administration, ALM201 was distributed from the anterior to the posterior segment of the eye with moderate-to-high relative abundance detected in the cornea, aqueous humor, lens, vitreous humor, and chorioretinal regions (Fig 3B). In ALM201-treated eyes, further analyses of various regions of interest (ROIs; Fig 3C1) of ALM201 peak intensities normalized by matrix cluster ion (Fig 3C2) and ROI area (Fig 3C3) showed that ALM201 intensities were detectable in all ROIs, with the highest ALM201 intensities observed in vitreous humor (ROI III) and retina and choroid (ROI V), respectively (Fig 3C1–3).

### Topical Application of ALM201 Suppresses CNV

**Figure 4:**
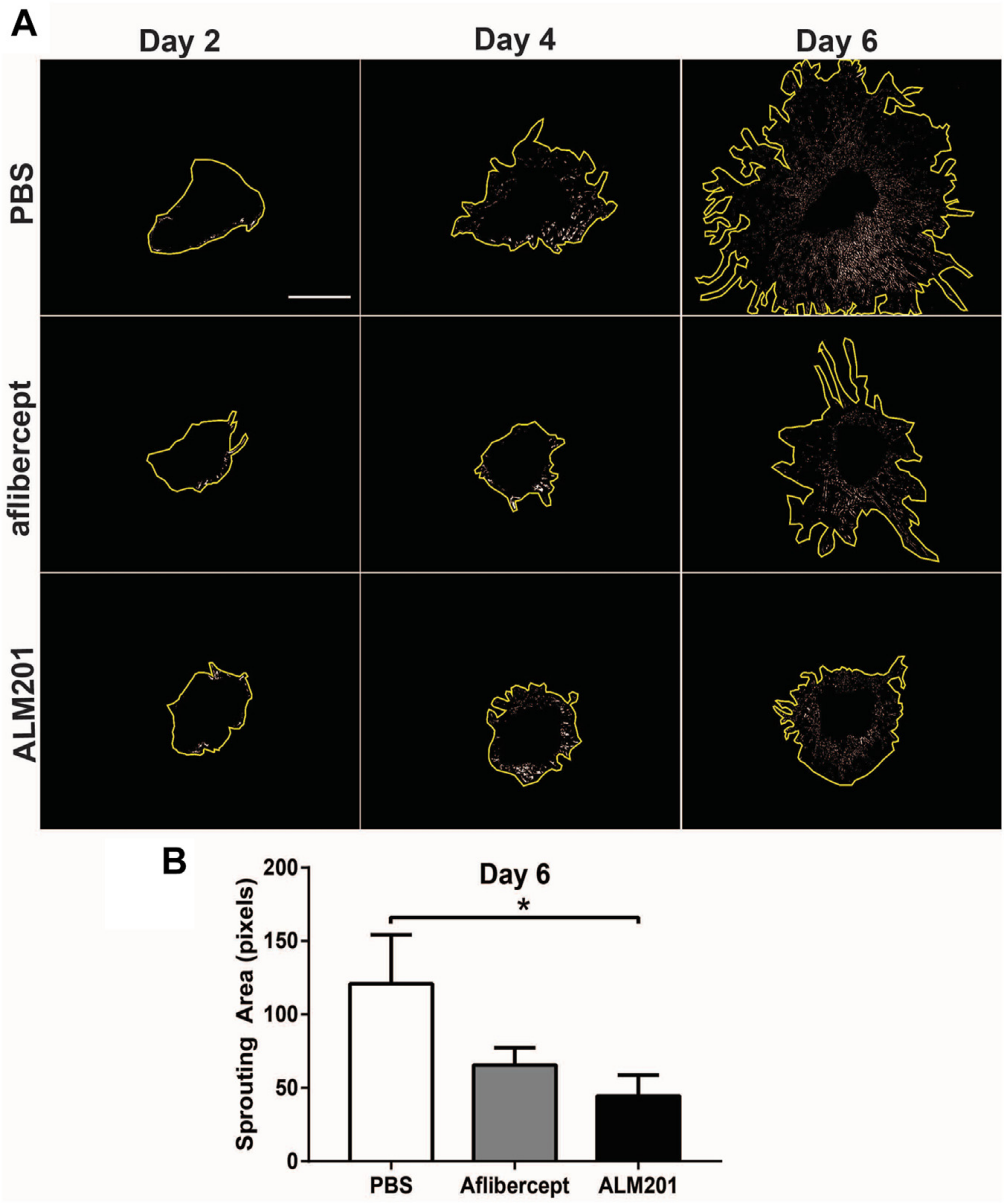
Antineovascularization effects of ALM201 in the choroid sprouting assay (CSA). **A**, Representative images showing effects of treatments with either phosphate-buffered saline (PBS), aflibercept, or ALM201 at days 2, 4, and 6 of explant culture. At day 6 of choroid culture and respective treatments, reduced choroid sprouting in ALM201 and aflibercept-treated CSA are visible. Yellow lines around sprouting area show maximum extent of sprouting. Scale bar, 1 mm. **B**, Graph showing the quantitation of choroidal sprouts at day 6 of culture. Choroid sprouting is reduced significantly with ALM201 treatment compared with vehicle (PBS) treatment. Results are presented as mean ± standard error of the mean (n = 7). ∗*P* < 0.05, 1-way analysis of variance.

The potential of ALM201 to treat CNV was assessed by analyzing choroidal sprouting area in the well-established choroidal sprouting assay model. At days 2 and 4 of explant culture, vessel sprouting area from choroid tissue explants was observed to be minimal (Fig 4A); however, by day 6 of explant culture, compared with other treatments, choroid sprouting was reduced in ALM201-treated choroid tissues (Fig 4A, B). Quantitation of choroid sprouting area showed that, by day 6 of treatment, ALM201 treatment (100 nM; 44.5 ± 14.31 pixels) significantly reduced choroid sprouting area compared with vehicle (PBS) treatment (Fig 4B; 120.9 ± 33.37 pixels; *P* = 0.04) and showed enhanced inhibition compared with the use of the anti-VEGF agent aflibercept (Fig 4B; 0.5 mg/ml; 65.63 ± 11.86 pixels; *P* = 0.7459).

### Topical Application of ALM201 Is Well Tolerated in an In Vivo Rat Model of nAMD

Results of the ocular slit-lamp examinations for local ocular tolerance are summarized in Table 1. Briefly, no adverse ocular signs related to topical ALM201 treatment were observed during the study; however, corneal opacity was observed with increased frequency and severity in the intravitreally administered aflibercept group. No ocular findings were observed in the untreated contralateral eyes of the animals in these groups. Additionally, no changes in body weight, general clinical signs, or systemic adverse effects were reported for any of the rats on the study. The details of ocular examinations by slit-lamp and scoring by the McDonald–Shadduck scale are provided in Supplemental Table S1 (S1A–S1D) and Supplemental Appendix 1.

**Table 1:** Local Tolerance Observations by Slit-lamp Evaluation

**Treatment**	**Adverse Sign**	**Day Observed**
Vehicle (topical PBS)	R#8 had mild (scored 1/4) corneal opacity covering up to 25% of the corneal area.	Day 3
Topical ALM201 (10 μM)	R#19 had an abnormal fundus related to laser-induced lesion surrounded by blood under the retina.	Day 20
IVT aflibercept (0.5 mg/mL)	R#60 had mild corneal opacity (scored 1/4) covering up to 25% of the corneal area.	Day 3
	R#60 had mild corneal opacity (scored 1/4) covering up to 50% of the corneal area.	Day 7 and 13
	R#60 had corneal opacity (scored 2/4) covering up to 50% of the corneal area.	Day 20
	R#61 had opacification of the posterior lens capsule (scored 1/1).	Days 7, 13, and 20
	R#57 had an abnormal fundus (scored 1/1).	Day 20

Local tolerance and adverse reactions were scored using the McDonald–Shadduck scale (*n* = 8/group). Baseline observations were made on day 0. Although multiple tissues were examined and adverse clinical signs were looked for, only adverse observations are reported above. Additional detail is provided in Table S2. IVT = intravitreal; PBS = phosphate-buffered saline; R = rat.

### Topical Application of ALM201 Reduces Vessel Leakiness in an In Vivo Rat Model of nAMD

**Figure 5:**
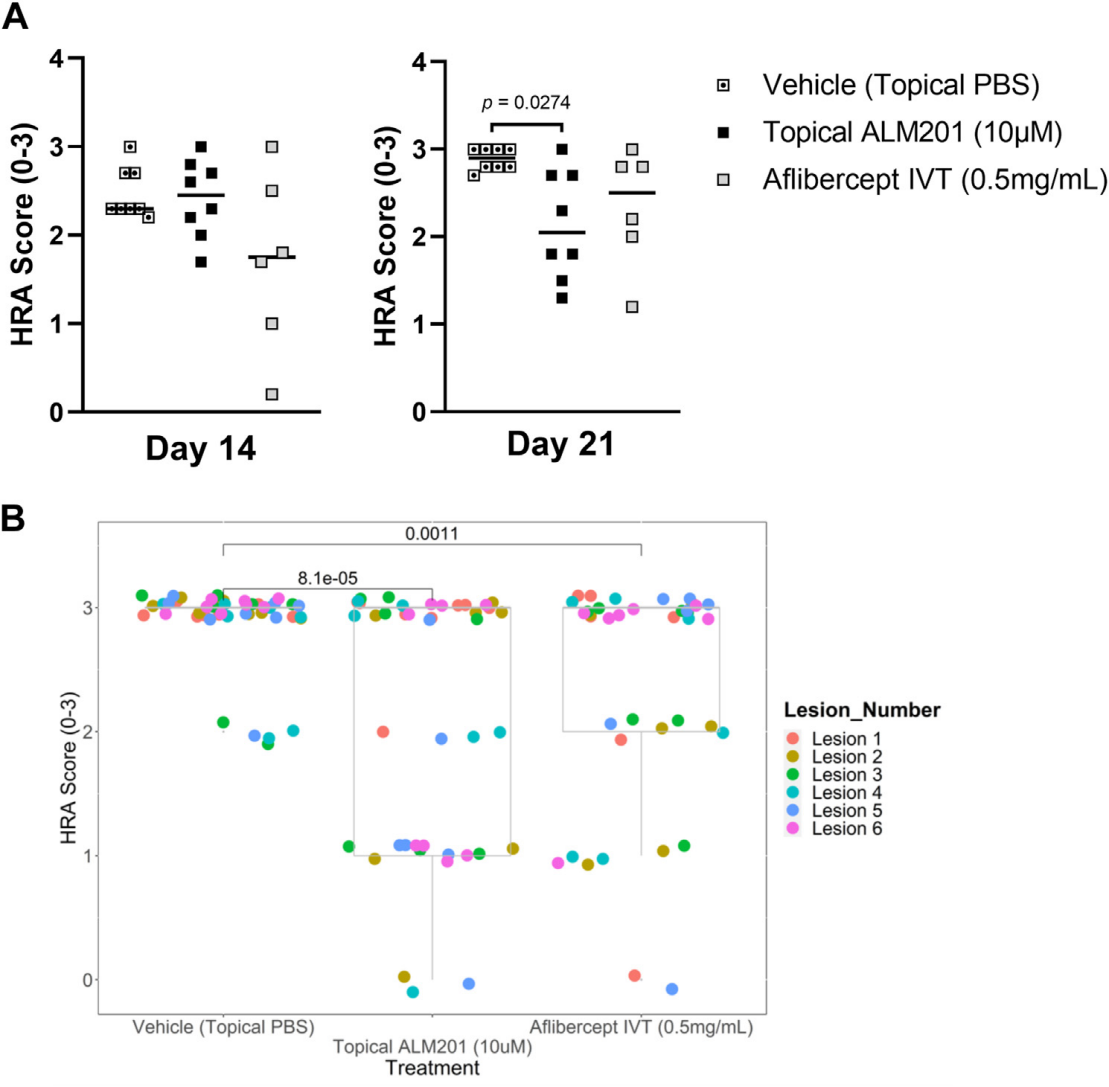
Angiographic evaluation scores of topical ALM201 administration versus topical phosphate-buffered saline (PBS) vehicle and intravitreal (IVT) aflibercept on day 21. **A**, Scatterplot showing the angiography score for each rat within a treatment group (symbols), along with the group median (horizontal bar). Each data point represents the mean of multiple evaluable lesions per rat. **B**, Scatterplot showing lesion-by-lesion analysis of data in (**A**). Each individual lesion score was plotted separately in scatterplot format, with each of the 6 lesions represented by a different color. Multiple spots of the same color represent the same lesion position in different rats within that treatment group. Only individual lesions that were not evaluable are excluded. Boxplot upper and lower hinges correspond to 25th and 75th percentiles, with the midpoint corresponding to the median. Whiskers extend 1.5 × interquartile range of the hinge. HRA = Heidelberg retinal angiograph.

The results of in-life angiographic evaluations with topical ALM201 versus a topical PBS vehicle control and intravitreal aflibercept are summarized in Table 2 and are illustrated as scatterplots in Figure 5A, B. Raw data for angiographic evaluations are provided in Supplemental Figure S2 and Supplemental Table S2 (S2A–S2C). Angiography revealed that topical ALM201 treatment significantly reduced the vessel leakiness score to less than that of the topical vehicle on day 21 (*P* = 0.0274, Dunn’s multiple comparison test of ALM201 vehicle topical vs. ALM201 10 μM topical; Fig 5A). This difference was confirmed with a lesion-by-lesion analysis (*P* = 8.1 × 10^–5^, Kruskal–Wallis test) and is illustrated in Figure 5B, where each of the 6 different colored symbols represent each of the 6 different lesions per rat eye. Interestingly, on day 14, early signs of efficacy were observed largely only in the intravitreal aflibercept group (twice weekly injections of 0.2 mg in 5 μl). When the median angiography scores were considered at the end of the dosing period (day 21), topical ALM201 delivered as strong a response, if not better, than aflibercept. This efficacious effect can be visualized readily in the lesion-by-lesion analysis showing that 14 lesions were scored 0 or 1 (highly efficacious effect) in the topical ALM201 treatment group, whereas only 8 lesions were scored as this effective in the intravitreal aflibercept group (Fig 5B); however, this difference was not found to be statistically significant (*P* = 0.39).

**Table 2:** Angiography Results on Days 14 and 21

**Treatment**	**Angiography Evaluation (**M**edian)**	
	**Day 14**	**Day 21**
Vehicle (topical PBS)	2.3 (*n* = 8/8)	2.9 (*n* = 8/8)
Topical ALM201 (10 μM)	2.4 (*n* = 8/8)	2.1 (*n* = 8/8); ***P*** **= 0.0274**
IVT aflibercept (0.5 mg/mL)	1.8 (*n* = 6/8)	2.5 (*n* = 6/8)

Note: *n* = number of animals that could be evaluated out of a group of 8; statistically significant effects (*p* < 0.05) of treatment versus topical PBS vehicle with Dunn’s test are shown in bold. IVT = intravitreal; PBS = phosphate-buffered saline.

### Topical Application of ALM201 Reduces Vascularized Lesion Size in an In Vivo Rat Model of nAMD

**Figure 6:**
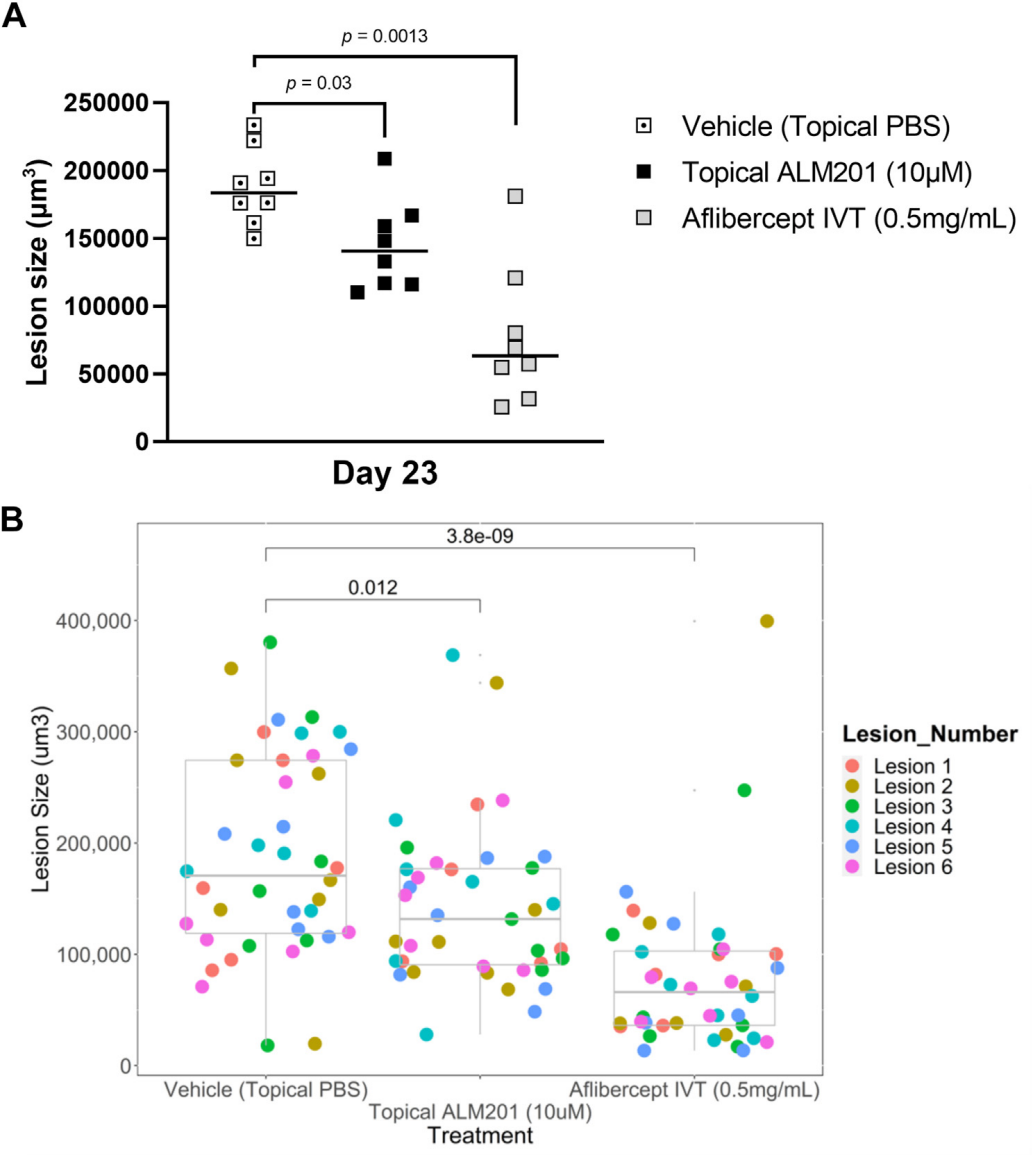
Individual choroidal neovascularization lesion sizes with topical ALM201 administration versus topical phosphate-buffered saline (PBS) vehicle and intravitreal (IVT) aflibercept on day 23. Lesion size (volume in cubic micrometers) was measured using the endothelial cell marker isolectin B4 on day 23 flat mounts. **A**, Scatterplot showing mean lesion volume for evaluable lesions from each rat plotted per rat. Group medians are represented by a horizontal line (n = 8 rats/group). Only statistically significant effects (Dunn’s test) are shown on the graph. **B**, Scatterplot showing lesion-by-lesion analysis. The volume of each individual lesion was plotted in scatterplot format; each 1 of 6 separate lesions per rat is plotted in a different color. Multiple spots of the same color represent the same lesion position in different rats within that treatment group. Only individual lesions that were not evaluable are excluded. Boxplot upper and lower hinges correspond to 25th and 75th percentiles, with the midpoint corresponding to the median. Whiskers extend 1.5 × interquartile range of the hinge. Only statistically significant effects (Wilcoxon ranked-sum test) are shown on the graph. Representative fluorescein isothiocyanate fluorescein isothiocyanate−stained flat-mount images are provided in Figure S3.

At the end of the study period (2 days after topical ALM201 dosing finished), rats were euthanized, and retinal choroid tissue was harvested to measure lesion size. Multiple flat-mount sections were stained with the endothelial cell marker isolectin B4, and z-stack images were collated to estimate lesion volume (in cubic micrometers). Topical ALM201 produced a statistically significant reduction in neovascularized lesion size, as did intravitreally dosed aflibercept (*P* = 0.03 and *P* = 0.0013, respectively, Dunn’s test). Lesion sizes at the end of the study are summarized as median data in Table 3 and as scatterplots in Figure 6A, B (Supplemental Fig S3 and Supplemental Table S3).

**Table 3:** Neovascularized Lesion Size on Day 23

Treatment	Median Lesion Size (μm^3^) on Day 23
Vehicle (topical PBS)	187 923 (n = 8/8)
Topical ALM201 (10 μM)	144 729 (n = 8/8); ***P* = 0.03**
IVT aflibercept (0.5 mg/ml)	63 137 (n = 8/8); ***P* = 0.0013**

Note: *n* = number of animals that could be evaluated (out of 8 animals in group); statistically significant effects (*p* < 0.05) with Dunn’s test versus topical PBS vehicle are shown in bold. IVT = intravitreal; PBS = phosphate-buffered saline.

## Discussion

Ocular neovascularization is central to various blinding conditions where effective, noninvasive treatments are still needed. Herein, we report, for the first time, the efficacy of topical ALM201 in effectively suppressing neovascularization in both anterior (cornea; in vivo, Fig 1) and posterior (choroid; ex vivo, Fig 4; in vivo, Fig 6) segments of the eye. We also showed that ALM201 treatment reduces cellular infiltration in the corneal stroma that is concomitant with inflammation (Fig 2). Furthermore, 1 hour after noninvasive, topical application to the eye, abundant regional biodistribution of ALM201 was found across all tissues of interest in the eye, from the cornea to the choroid (Fig 3). This suggests that ALM201 may be a potent therapeutic candidate for vascular conditions of the eye, including CV, nAMD, proliferative diabetic retinopathy, retinopathy of prematurity, and other ocular anomalies where vascularization is implicated.

After corneal injury, an upregulation of proinflammatory cytokines and growth factors occurs that triggers infiltration of leukocytes, including neutrophils, lymphocytes, and macrophages.^6^^,^^21^^,^^33^ Approximately 12 to 24 hours after corneal injury, leukocytes are considered to infiltrate from the limbal blood vessels and possibly the tear film into the corneal stroma, and this infiltration typically is accompanied by an angiogenic response.^6^^,^^33^ Furthermore, corneal stroma keratocytes, which normally are quiescent physiologically, assume activated phenotypes in pathologic conditions and migrate to sites of corneal injury.^6^^,^^21^^,^^33^ In line with this, the marked suppression of corneal stroma cellular infiltration reported in this study (Fig 2C) suggests that ALM201 reduces corneal inflammation by inhibiting cellular infiltration into the sites of corneal wounding. Also, in addition to reducing corneal neovascularization and cellular infiltration dramatically, clinical indicators of corneal inflammation, such as opacity or whiteness and edema, were also reduced in ALM201-treated eyes (Fig 1B).

Leukocytes are known to play a critical role in CNV, contributing to nAMD via their role in elevating proangiogenic cytokines and growth factors, including vascular endothelial growth factor. Subsequent breakdown of the blood–retinal barrier and sprouting of fragile blood vessels from the choroid into the sub–retinal pigment epithelium or subretina of the macula cause edema and acute vision loss.^5^^,^^34^ Because CNV is central to nAMD, significant reduction of CNV by pharmacologic intervention of ALM201 suggests that ALM201 has the potential to treat nAMD. The choroidal sprouting assay is an ex vivo model of microvascular angiogenesis that is well characterized, standardized, and reproducible.^29^ After treatment with an anti-VEGF agent (aflibercept) at the recommended clinical dose (0.5 mg/ml) in an ex vivo choroidal sprouting assay, we did not find its antineovascularization effects to be as effective as that of ALM201 in this assay system (Fig 3B). This further supports the therapeutic antineovascularization potential of ALM201. The effectiveness of topical ALM201 on neovascularization in the posterior eye tissue along with rapid and wide biodistribution of ALM201 in the posterior tissues of the eye has potentially important clinical implications because of the obvious advantages of administering ALM201 topically over current invasive intraocular treatments.

It should be highlighted that once daily topical administrations of ALM201 provided significant benefit in this experimental model of nAMD, reducing both vessel leakiness and lesion size compared with vehicle-treated animals (Figs 5 and 6). Interestingly, topical ALM201 reduced vessel leakiness to a greater extent than intravitreal aflibercept, although this did not translate to a smaller lesion volume. This may relate directly to the putative mechanism of action of ALM201, where its effects on endothelial cell biology and vessel integrity would be important in terms of upstream pathologic features to target at an early stage of nAMD.^35^ Alternatively, it may reflect a limitation of the dose, duration of dosing, or limitations of the model itself (i.e., induction of neovascular lesions with a laser as opposed to de novo disease). Regardless, to achieve this level of efficacy with daily administrations of low micromolar doses in a simple, nonoptimized formulation provides a very strong basis on which to develop ALM201 topically further for eye conditions. Furthermore, although this is the first demonstration of efficacy for ALM201 in an in vivo proof-of-concept nAMD model, the potential also exists to deliver ALM201 as a maintenance therapy between intravitreal doses of anti-VEGF agents such as aflibercept, where it may be possible to reduce the number or frequency of intravitreal injections or both. A concomitant benefit in delivering ALM201 throughout the eye seems to be a reduction in associated inflammation and opacity and reduced edema, as observed by slit-lamp examination. Although a formal toxicology assessment (including an evaluation of photoreceptors, retinal ganglion cells, and retinal function) would be required before entering clinical trials, the current proof-of-concept safety profile of ALM201, particularly the lack of systemic adverse effects and excellent ocular tolerability, further support clinical development. A dual anti-inflammatory and antiangiogenic mechanism of action would be of value to patients with nAMD and would be a valuable clinical therapeutic strategy.

In addition to AMD, diseases that affect the posterior segment of the eye that also are leading causes of vision loss include diabetic retinopathy, diabetic macular edema, and proliferative vitreoretinopathy, conditions that urgently require noninvasive therapeutic alternatives.^36^ Although the anterior of the eye, including the cornea, is easily accessible for noninvasive drug delivery, a major challenge for the treatment of diseases of the posterior segment of the eye, including the retina and choroid, is that it is not easily accessible for topical delivery of treatments because of its complexity and the various physiologic and anatomic barriers that impede drug biodistribution to the intended ocular destination.^36^ Regardless of these challenges, topical eye drops remain a preferred route of ocular drug administration because they are noninvasive, are more convenient to use, have higher rates of patient compliance, and permit simultaneous lower total drug dosage with high local concentrations.^27^^,^^36^

ALM201, a small, 23-amino acid synthetic peptide derived from a naturally occurring human protein that is neither immunogenic nor cytotoxic, yet has potent anti-inflammatory and antiangiogenic activity, is an ideal candidate for therapeutic repurposing from oncology to ophthalmology. From our repurposing investigations—verification of the colocation of ALM201 with relevant posterior ocular landmarks, especially the retina and choroid, 1 hour after topical eye drop application—we demonstrated via in vivo proof-of-concept preclinical studies that ALM201 can be delivered topically effectively and can reach the posterior of the eye within a short period. Importantly, ALM201 was well tolerated, and neither local nor systemic adverse effects were observed in our preclinical ophthalmology studies. This is supported further by safety data from a phase Ia clinical trial in which ALM201 was dosed systemically to patients with solid tumors at doses of up to 300 mg per patient (maximum administrable dose).^25^^,^^26^ These doses were well tolerated and confirmed the excellent safety profile of ALM201 in prior nonclinical good laboratory practice safety studies. When considered together, the low doses required to achieve efficacy in the preclinical models, the topical eye drop route of administration, the lack of cytotoxicity and immunogenicity attributed to ALM201, and its excellent efficacy for anti-inflammatory and antiangiogenic activity in the eye make ALM201 an ideal candidate for repurposing as a topical treatment for ocular diseases.

The development of a new prescription drug from discovery to market authorization costs, on average, > $2.5 billion United States dollars^37^ and can take up to 12 years or more.^38^ Repurposing drugs that have been shown to be safe in clinical trials significantly alleviates time and cost burdens and often expedites delivery of new treatments to patients who urgently need them. The antineovascularization activity of ALM201 has been reported in the context of oncology with advances made to first-in-human clinical trials.^25^^,^^26^ Based on our results and the promising safety profile of ALM201 in patients with ovarian cancer and other solid tumors, the potential of ALM201 to inhibit pathologic vascularization effectively in the clinical ophthalmology setting is encouraging; however, the mechanism of action of ALM201 currently is not fully understood. Previous studies reported it to have inhibitory activity against multiple kinases implicated in angiogenesis and to target the CD44 pathway,^23^^,^^24^ which is purportedly inflammatory,^39^ as well as affecting endothelial cell migration. These findings suggest that pleiotropic biological effects and polypharmacologic factors are likely. Future investigations not only will validate the outcomes of this study but also likely will elucidate further the direct targets, the mechanism of action on multiple biological pathways, the ocular prophylactic and toxicological profiles, the ocular pharmacokinetics and residency times, and the optimum doses and schedules in which to deliver maximum therapeutic benefit to patients with debilitating ocular diseases.

## Conclusions

We have shown that, when delivered topically to the eye, ALM201 impedes pathologic ocular neovascularization, reduces inflammation, demonstrates rapid penetration and wide ocular biodistribution, and offers promising therapeutic efficacy for treating anterior and posterior ocular disease with underlying vascular pathologic features. Current management of neovascular ocular conditions urgently needs effective alternative therapeutic approaches. ALM201 has been proven to be safe in early clinical trials for solid tumors and warrants further investigation for its preventative or therapeutic potential in the clinical management of sight-limiting neovascular ocular conditions.

## Supplementary Data

Table S1A

Table S1B

Table S1C

Table S1D

Table S2A-C

## References

[1] Folkman J., Shing Y. (1992). Angiogenesis. J Biol Chem.

[2] Salajegheh A. (2016). Angiogenesis in Health, Disease and Malignancy.

[3] Felmeden D.C., Blann A.D., Lip G.Y.H. (2003). Angiogenesis: basic pathophysiology and implications for disease. Eur Heart J.

[4] Qazi Y., Maddula S., Ambati B.K. (2009). Mediators of ocular angiogenesis. J Genet.

[5] Chen M., Xu H. (2015). Parainflammation, chronic inflammation, and age-related macular degeneration. J Leukoc Biol.

[6] Wilson S.E., Mohan R.R., Mohan R.R. (2001). The corneal wound healing response: cytokine-mediated interaction of the epithelium, stroma, and inflammatory cells. Prog Retin Eye Res.

[7] Pascolini D., Mariotti S.P. (2012). Global estimates of visual impairment: 2010. Br J Ophthalmol.

[8] Bressler N.M., Bressler S.B., Fine S.L. (1988). Age-related macular degeneration. Surv Ophthalmol.

[9] Kaiser P.K. (2008). Ranibizumab: the evidence of its therapeutic value in neovascular age-related macular degeneration. Core Evid.

[10] Patel K.H., Chow C.C., Rathod R. (2013). Rapid response of retinal pigment epithelial detachments to intravitreal aflibercept in neovascular age-related macular degeneration refractory to bevacizumab and ranibizumab. Eye.

[11] Schmid M.K., Bachmann L.M., Fäs L. (2015). Efficacy and adverse events of aflibercept, ranibizumab and bevacizumab in age-related macular degeneration: a trade-off analysis. Br J Ophthalmol.

[12] Azar D.T. (2006). Corneal angiogenic privilege: angiogenic and antiangiogenic factors in corneal avascularity, vasculogenesis, and wound healing (an American Ophthalmological Society thesis). Trans Am Ophthalmol Soc.

[13] Chang J.H., Gabison E.E., Kato T., Azar D.T. (2001). Corneal neovascularization. Curr Opin Ophthalmol.

[14] Gupta D., Illingworth C. (2011). Treatments for corneal neovascularization: a review. Cornea.

[15] Feizi S., Azari A.A., Safapour S. (2017). Therapeutic approaches for corneal neovascularization. Eye Vis.

[16] Razeghinejad M.R., Katz L.J. (2012). Steroid-induced iatrogenic glaucoma. Ophthalmic Res.

[17] Kersey J.P., Broadway D.C. (2006). Corticosteroid-induced glaucoma: a review of the literature. Eye (Lond).

[18] Sarnicola V., Toro P. (2010). Deep anterior lamellar keratoplasty in herpes simplex corneal opacities. Cornea.

[19] Feizi S., Simionescu D., Simionescu A. (2017). Physiologic and Pathologic Angiogenesis-Signaling Mechanisms and Targeted Therapy.

[20] Bachmann B., Taylor R.S., Cursiefen C. (2010). Corneal neovascularization as a risk factor for graft failure and rejection after keratoplasty: an evidence-based meta-analysis. Ophthalmology.

[21] Yu H., Sun L., Cui J. (2019). Three kinds of corneal host cells contribute differently to corneal neovascularization. EBioMedicine.

[22] Robson T., James I.F. (2012). The therapeutic and diagnostic potential of FKBPL; a novel anticancer protein. Drug Discov Today.

[23] Valentine A., O’Rourke M., Yakkundi A. (2011). FKBPL and peptide derivatives: novel biological agents that inhibit angiogenesis by a CD44-dependent mechanism. Clin Cancer Res.

[24] McClements L., Yakkundi A., Papaspyropoulos A. (2013). Targeting treatment-resistant breast cancer stem cells with FKBPL and its peptide derivative, AD-01, via the CD44 pathway. Clin Cancer Res.

[25] El Helali, A, Plummer, R, Jayson, GC, et al. A first-in-human phase I dose-escalation trial of the novel therapeutic peptide, ALM201, demonstrates a favourable safety profile in unselected patients with ovarian cancer and other advanced solid tumours. *Br Cancer,* In press. 10.1038/s41416-022-01780-z PMC9276671 35568736

[26] EU Clinical Trials Register A phase I open-label multicentre dose-escalation study of subcutaneous ALM201 in patients with advanced ovarian cancer and other solid tumours. https://www.clinicaltrialsregister.eu/ctr-search/trial/2014-001175-31/GB.

[27] Grove K.J., Kansara V., Prentiss M. (2017). Application of imaging mass spectrometry to assess ocular drug transit. SLAS Discov Adv Life Sci RD.

[28] Mori N., Mochizuki T., Yamazaki F. (2019). MALDI imaging mass spectrometry revealed atropine distribution in the ocular tissues and its transit from anterior to posterior regions in the whole-eye of rabbit after topical administration. PloS One.

[29] Shao Z., Friedlander M., Hurst C.G. (2013). Choroid sprouting assay: an ex vivo model of microvascular angiogenesis. PloS One.

[30] Tucker L.H., Conde-González A., Cobice D. (2018). MALDI matrix application utilizing a modified 3D printer for accessible high resolution mass spectrometry imaging. Anal Chem.

[31] McDonald T.O., Shadduck J.A., Marzulli F.N., Maibach H.I. (1977). Dermato Toxicology and Pharmacology. Vol 4. Advances in Modern Toxicology.

[32] Baldwin H.A., McDonald T.O., Beasley C.H. (1973). Slit-lamp examination of experimental animal eyes. II. Grading scales and photographic evaluation of induced pathological conditions. J Soc Cosmet Chem.

[33] O’Brien T.P., Li Q., Ashraf M.F. (1998). Inflammatory response in the early stages of wound healing after excimer laser keratectomy. Arch Ophthalmol.

[34] Kauppinen A., Paterno J.J., Blasiak J. (2016). Inflammation and its role in age-related macular degeneration. Cell Mol Life Sci.

[35] Swaminathan S., Cranston A.N., Clyne A.M. (2019). A three-dimensional in vitro coculture model to quantify breast epithelial cell adhesion to endothelial cells. Tissue Eng Part C Methods.

[36] Hughes P.M., Olejnik O., Chang-Lin J.-E., Wilson C.G. (2005). Topical and systemic drug delivery to the posterior segments. Adv Drug Deliv Rev.

[37] DiMasi J.A., Grabowski H.G., Hansen R.W. (2016). Innovation in the pharmaceutical industry: new estimates of R&D costs. J Health Econ.

[38] Van Norman G.A. (2016). Drugs, devices, and the FDA: part 1: an overview of approval processes for drugs. JACC Basic Transl Sci.

[39] Puré E., Cuff C.A. (2001). A crucial role for CD44 in inflammation. Trends Mol Med.

