# Supplementary material for: Successful Proof-of-Concept for Topical Delivery of Novel Peptide ALM201 with Potential Usefulness for Treating Neovascular Eye Disorders
Source: Ophthalmol Sci. 2022 Apr 4;2(2):100150. doi: 10.1016/j.xops.2022.100150 (PMC9560569; doi:10.1016/j.xops.2022.100150)
Supplement: Table S1A [file mmc1.pdf]

Topical Vehicle (PBS)

	CONJUNCTIVA																			
	Congestion (0-3)						Swelling (0-4)								Discharge (0-3)					
	Right eye			Left eye			Right eye				Left eye				Right eye			Left eye		
**DAY OF STUDY**	1	2	3	1	2	3	1	2	3	4	1	2	3	4	1	2	3	1	2	3
**Baseline**	-	-	-	-	-	-	-	-	-	-	-	-	-	-	-	-	-	-	-	-
**D3**	-	-	-	-	-	-	-	-	-	-	-	-	-	-	-	-	-	-	-	-
**D7**	-	-	-	-	-	-	-	-	-	-	-	-	-	-	-	-	-	-	-	-
**D13**	-	-	-	-	-	-	-	-	-	-	-	-	-	-	-	-	-	-	-	-
**D20**	-	-	-	-	-	-	-	-	-	-	-	-	-	-	-	-	-	-	-	-

	CORNEA																			
	Degree of opacity (0-4)								Area of opacity (0-4)								Pannus (0-2)			
	Right eye				Left eye				Right eye				Left eye				Right eye		Left eye	
**DAY OF STUDY**	1	2	3	4	1	2	3	4	1	2	3	4	1	2	3	4	1	2	1	2
**Baseline**	-	-	-	-	-	-	-	-	-	-	-	-	-	-	-	-	-	-	-	-
**D3**	R#8	-	-	-	-	-	-	-	R#8	-	-	-	-	-	-	-	-	-	-	-
**D7**	-	-	-	-	-	-	-	-	-	-	-	-	-	-	-	-	-	-	-	-
**D13**	-	-	-	-	-	-	-	-	-	-	-	-	-	-	-	-	-	-	-	-
**D20**	-	-	-	-	-	-	-	-	-	-	-	-	-	-	-	-	-	-	-	-

	CORNEA																AQUEOUS FLARE						IRIS							
	Intensity of staining (0-4)								Area of staining (0-4)								Tyndall (0-3)						Hyperhemia (0-4)							
	Right eye				Left eye				Right eye				Left eye				Right eye			Left eye			Right eye				Left eye			
**DAY OF STUDY**	1	2	3	4	1	2	3	4	1	2	3	4	1	2	3	4	1	2	3	1	2	3	1	2	3	4	1	2	3	4
**Baseline**	-	-	-	-	-	-	-	-	-	-	-	-	-	-	-	-	-	-	-	-	-	-	-	-	-	-	-	-	-	-
**D3**	-	-	-	-	-	-	-	-	-	-	-	-	-	-	-	-	-	-	-	-	-	-	-	-	-	-	-	-	-	-
**D7**	-	-	-	-	-	-	-	-	-	-	-	-	-	-	-	-	-	-	-	-	-	-	-	-	-	-	-	-	-	-
**D13**	-	-	-	-	-	-	-	-	-	-	-	-	-	-	-	-	-	-	-	-	-	-	-	-	-	-	-	-	-	-
**D20**	-	-	-	-	-	-	-	-	-	-	-	-	-	-	-	-	-	-	-	-	-	-	-	-	-	-	-	-	-	-

	LENS (0/1)		FUNDUS (0/1)	
	**Right eye**	**Left eye**	**Right eye**	**Left eye**
**DAY OF STUDY**	**1**	**1**	**1**	**1**
**Baseline**	-	-	-	-
**D3**	-	-	-	-
**D7**	-	-	-	-
**D13**	-	-	-	-
**D20**	-	-	-	-

Table S1A: Ocular examinations by slit-lamp and scoring by McDonald-Shadduck scales for rats in the topical vehicle (PBS) group. A dash (-) = nothing observed. R# = rat number.
