# Supplementary material for: Successful Proof-of-Concept for Topical Delivery of Novel Peptide ALM201 with Potential Usefulness for Treating Neovascular Eye Disorders
Source: Ophthalmol Sci. 2022 Apr 4;2(2):100150. doi: 10.1016/j.xops.2022.100150 (PMC9560569; doi:10.1016/j.xops.2022.100150)
Supplement: Table S1B [file mmc2.pdf]

Topical ALM201 (10 µM)

	CONJUNCTIVA																			
	**Congestion (0-3)**						**Swelling (0-4)**								**Discharge (0-3)**					
	Right eye			Left eye			Right eye				Left eye				Right eye			Left eye		
**DAY OF STUDY**	1	2	3	1	2	3	1	2	3	4	1	2	3	4	1	2	3	1	2	3
**Baseline**	-	-	-	-	-	-	-	-	-	-	-	-	-	-	-	-	-	-	-	-
**D3**	-	-	-	-	-	-	-	-	-	-	-	-	-	-	-	-	-	-	-	-
**D7**	-	-	-	-	-	-	-	-	-	-	-	-	-	-	-	-	-	-	-	-
**D13**	-	-	-	-	-	-	-	-	-	-	-	-	-	-	-	-	-	-	-	-
**D20**	-	-	-	-	-	-	-	-	-	-	-	-	-	-	-	-	-	-	-	-

	CORNEA																			
	**Degree of opacity (0-4)**								**Area of opacity (0-4)**								**Pannus (0-2)**			
	Right eye				Left eye				Right eye				Left eye				Right eye		Left eye	
**DAY OF STUDY**	1	2	3	4	1	2	3	4	1	2	3	4	1	2	3	4	1	2	1	2
**Baseline**	-	-	-	-	-	-	-	-	-	-	-	-	-	-	-	-	-	-	-	-
**D3**	-	-	-	-	-	-	-	-	-	-	-	-	-	-	-	-	-	-	-	-
**D7**	-	-	-	-	-	-	-	-	-	-	-	-	-	-	-	-	-	-	-	-
**D13**	-	-	-	-	-	-	-	-	-	-	-	-	-	-	-	-	-	-	-	-
**D20**	-	-	-	-	-	-	-	-	-	-	-	-	-	-	-	-	-	-	-	-

	CORNEA																AQUEOUS FLARE						IRIS							
	**Intensity of staining (0-4)**								**Area of staining (0-4)**								**Tyndall (0-3)**						**Hyperhemia (0-4)**							
	Right eye				Left eye				Right eye				Left eye				Right eye			Left eye			Right eye				Left eye			
**DAY OF STUDY**	1	2	3	4	1	2	3	4	1	2	3	4	1	2	3	4	1	2	3	1	2	3	1	2	3	4	1	2	3	4
**Baseline**	-	-	-	-	-	-	-	-	-	-	-	-	-	-	-	-	-	-	-	-	-	-	-	-	-	-	-	-	-	-
**D3**	-	-	-	-	-	-	-	-	-	-	-	-	-	-	-	-	-	-	-	-	-	-	-	-	-	-	-	-	-	-
**D7**	-	-	-	-	-	-	-	-	-	-	-	-	-	-	-	-	-	-	-	-	-	-	-	-	-	-	-	-	-	-
**D13**	-	-	-	-	-	-	-	-	-	-	-	-	-	-	-	-	-	-	-	-	-	-	-	-	-	-	-	-	-	-
**D20**	-	-	-	-	-	-	-	-	-	-	-	-	-	-	-	-	-	-	-	-	-	-	-	-	-	-	-	-	-	-

	LENS		FUNDUS	
	**(0/1)**		**(0/1)**	
	**Right eye**	**Left eye**	**Right eye**	**Left eye**
**DAY OF STUDY**	**1**	**1**	**1**	**1**
**Baseline**	-	-	-	-
**D3**	-	-	-	-
**D7**	-	-	-	-
**D13**	-	-	-	-
**D20**	-	-	R#19	-

Table S1B: Ocular examinations by slit-lamp and scoring by McDonald-Shadduck scales for rats in the topical ALM201 (10 µM) group. A dash (-) = nothing observed. R# = rat number.
