# Supplementary material for: Successful Proof-of-Concept for Topical Delivery of Novel Peptide ALM201 with Potential Usefulness for Treating Neovascular Eye Disorders
Source: Ophthalmol Sci. 2022 Apr 4;2(2):100150. doi: 10.1016/j.xops.2022.100150 (PMC9560569; doi:10.1016/j.xops.2022.100150)
Supplement: Table S1C [file mmc3.pdf]

IVT aflibercept (0.5 mg/mL)

	CONJUNCTIVA																			
	Congestion (0-3)						Swelling (0-4)								Discharge (0-3)					
	Right eye			Left eye			Right eye				Left eye				Right eye			Left eye		
**DAY OF STUDY**	1	2	3	1	2	3	1	2	3	4	1	2	3	4	1	2	3	1	2	3
**Baseline**	-	-	-	-	-	-	-	-	-	-	-	-	-	-	-	-	-	-	-	-
**D3**	-	-	-	-	-	-	-	-	-	-	-	-	-	-	-	-	-	-	-	-
**D7**	-	-	-	-	-	-	-	-	-	-	-	-	-	-	-	-	-	-	-	-
**D13**	-	-	-	-	-	-	-	-	-	-	-	-	-	-	-	-	-	-	-	-
**D20**	-	-	-	-	-	-	-	-	-	-	-	-	-	-	-	-	-	-	-	-

	CORNEA																			
	Degree of opacity (0-4)								Area of opacity (0-4)								Pannus (0-2)			
	Right eye				Left eye				Right eye				Left eye				Right eye		Left eye	
**DAY OF STUDY**	1	2	3	4	1	2	3	4	1	2	3	4	1	2	3	4	1	2	1	2
**Baseline**	-	-	-	-	-	-	-	-	-	-	-	-	-	-	-	-	-	-	-	-
**D3**	R#60	-	-	-	-	-	-	-	R#60	-	-	-	-	-	-	-	-	-	-	-
**D7**	R#60	-	-	-	-	-	-	-	-	R#60	-	-	-	-	-	-	-	-	-	-
**D13**	R#60	-	-	-	-	-	-	-	-	R#60	-	-	-	-	-	-	-	-	-	-
**D20**	-	R#60	-	-	-	-	-	-	-	R#60	-	-	-	-	-	-	-	-	-	-

	CORNEA																AQUEOUS FLARE						IRIS							
	Intensity of staining (0-4)								Area of staining (0-4)								Tyndall (0-3)						Hyperhemia (0-4)							
	Right eye				Left eye				Right eye				Left eye				Right eye			Left eye			Right eye				Left eye			
**DAY OF STUDY**	1	2	3	4	1	2	3	4	1	2	3	4	1	2	3	4	1	2	3	1	2	3	1	2	3	4	1	2	3	4
**Baseline**	-	-	-	-	-	-	-	-	-	-	-	-	-	-	-	-	-	-	-	-	-	-	-	-	-	-	-	-	-	-
**D3**	-	-	-	-	-	-	-	-	-	-	-	-	-	-	-	-	-	-	-	-	-	-	-	-	-	-	-	-	-	-
**D7**	-	-	-	-	-	-	-	-	-	-	-	-	-	-	-	-	-	-	-	-	-	-	-	-	-	-	-	-	-	-
**D13**	-	-	-	-	-	-	-	-	-	-	-	-	-	-	-	-	-	-	-	-	-	-	-	-	-	-	-	-	-	-
**D20**	-	-	-	-	-	-	-	-	-	-	-	-	-	-	-	-	-	-	-	-	-	-	-	-	-	-	-	-	-	-

	LENS				FUNDUS			
	(0/1)				(0/1)			
	Right eye		Left eye		Right eye		Left eye	
**DAY OF STUDY**	0	1	0	1	0	1	0	1
**Baseline**	-	-	-	-	-	-	-	-
**D3**	-	-	-	-	-	-	-	-
**D7**	-	R#61	-	-	-	-	-	-
**D13**	-	R#61	-	-	-	-	-	-
**D20**	-	R#61	-	-	-	R#57	-	-

Table S1C: Ocular examinations by slit-lamp and scoring by McDonald-Shadduck scales for rats in the IVT aflibercept (0.5 mg/mL) group. A dash (-) = nothing observed. R# = rat number.
