# Supplementary material for: Successful Proof-of-Concept for Topical Delivery of Novel Peptide ALM201 with Potential Usefulness for Treating Neovascular Eye Disorders
Source: Ophthalmol Sci. 2022 Apr 4;2(2):100150. doi: 10.1016/j.xops.2022.100150 (PMC9560569; doi:10.1016/j.xops.2022.100150)
Supplement: Table S1D [file mmc4.pdf]

Treatment	Rat n°	Day of study	Right eye	Left eye
ALM201 Vehicle topical	R#1, R#2, R#3, R#4, R#5, R#6, R#7, R#8	Baseline	-	-
		D3 to D20		
ALM201 10 µM topical	R#17, R#18, R#20, R#21, R#22, R#23, R#24	Baseline	-	-
		D3 to D20		
	R#19	Baseline	-	-
		D3		
		D7 to D20	Spot laser surrounded by blood under the retina	
Intravitreal aflibercept	R#57	Baseline	-	-
		D3 to D7	Spot laser surrounded by blood under the retina	
		D13	-	
		D20	Haemorrhage retinal, impact retinal, blood in vitreous	
	R#58, R#59	Baseline	-	-
		D3 to D20	Circle observed at the level of lens	Circle observed at the level of lens
	R#60	Baseline	-	-
		D3 to D7		
		D13	Partial mydriasis	
		D20	Shining particles in posterior capsule of lens	
	R#61	Baseline	-	-
		D3	Spot laser surrounded by little blood.	
		D7	Opacity in large quantity of posterior capsule of lens. Fundus not observed	
		D13 to D20	Opacity in large quantity of posterior capsule of lens. Fundus not observed partial mydriasis	
	R#62	Baseline	-	-
		D3 to D20		
	R#63	Baseline	-	-
		D3 to D7	Circle observed at the level of lens. Spot laser surrounded by little blood under the retina	Circle observed at the level of lens. Spot laser surrounded by little blood under the retina
		D13	Circle observed at the level of lens	
		D20	Circle observed at the level of lens	Circle observed at the level of lens only
	R#64	Baseline	-	-
		D3 to D13	Circle observed at the level of lens.<br>2 Spots lasers surrounded by little blood under the retina	Circle observed at the level of lens
		D20	Circle observed at the level of lens	

Table S1D: Additional comments on ocular examinations by slit-lamp for rats in the treatment groups. A dash (-) = nothing observed. R# = rat number.
