# Supplementary material for: Successful Proof-of-Concept for Topical Delivery of Novel Peptide ALM201 with Potential Usefulness for Treating Neovascular Eye Disorders
Source: Ophthalmol Sci. 2022 Apr 4;2(2):100150. doi: 10.1016/j.xops.2022.100150 (PMC9560569; doi:10.1016/j.xops.2022.100150)
Supplement: Table S2A-C [file mmc5.pdf]

A) Topical vehicle (PBS)

Time-point after induction	Eye	Rat n°	HRA evaluation 10 minutes after fluorescein injection (scale: 0-3 arbitrary unit)											
			Lesion N°						Individual Mean	Individual SD	*Mean*	*SD*	*MEDIAN*	Interquartile range
			1	*2*	*3*	*4*	*5*	*6*						
Day 14	R	R#1	3	2	1	2	3	3	**2.3**	0.8	***2.5***	*0.3*	***2.3***	*0.3*
		R#2	3	1	2	3	2	3	**2.3**	0.8				
		R#3	3	1	3	3	3	3	**2.7**	0.8				
		R#4	3	3	1	0	3	3	**2.2**	1.3				
		R#5	3	3	ND	ND	3	3	**3.0**	0.0				
		R#6	3	3	1	1	3	3	**2.3**	1.0				
		R#7	3	3	3	1	3	3	**2.7**	0.8				
		R#8	3	2	1	3	2	3	**2.3**	0.8				
Day 21	R	R#1	3	3	2	2	3	3	**2.7**	0.5	***2.9***	*0.1*	***2.9***	*0.2*
		R#2	3	3	3	3	3	3	**3.0**	0.0				
		R#3	3	3	3	3	2	3	**2.8**	0.4				
		R#4	3	3	2	3	3	3	**2.8**	0.4				
		R#5	3	3	ND	ND	3	3	**3.0**	0.0				
		R#6	3	3	3	3	3	3	**3.0**	0.0				
		R#7	3	3	3	2	3	3	**2.8**	0.4				
		R#8	3	3	3	3	3	3	**3.0**	0.0				

B) Topical ALM201 (10 µM)

Time-point after induction	Eye	Rat n°	HRA evaluation 10 minutes after fluorescein injection (scale: 0-3 arbitrary unit)											
			Lesion N°						Individual Mean	Individual SD	*Mean*	*SD*	*MEDIAN*	Interquartile range
			1	2	3	4	5	6						
Day 14	R	R#17	3	3	0	2	1	1	**1.7**	1.2	***2.4***	*0.4*	***2.4***	*0.6*
		R#18	3	3	2	2	ND	3	**2.6**	0.5				
		R#19	3	ND	ND	2	3	3	**2.8**	0.5				
		R#20	3	3	3	2	2	3	**2.7**	0.5				
		R#21	3	1	1	2	3	3	**2.2**	1.0				
		R#22	2	1	2	3	3	1	**2.0**	0.9				
		R#23	3	1	3	ND	ND	2	**2.3**	1.0				
		R#24	3	3	3	ND	ND	3	**3.0**	0.0				
Day 21	R	R#17	3	3	1	3	1	3	**2.3**	1.0	***2.1***	*0.6*	***2.0***	*1.0*
		R#18	3	1	1	0	0	3	**1.3**	1.4				
		R#19	3	ND	ND	2	1	1	**1.8**	1.0				
		R#20	3	3	3	2	2	3	**2.7**	0.5				
		R#21	2	1	1	3	1	1	**1.5**	0.8				
		R#22	3	3	3	3	3	1	**2.7**	0.8				
		R#23	3	0	3	ND	ND	1	**1.8**	1.5				
		R#24	3	3	3	ND	ND	3	**3.0**	0.0				

C) IVT aflibercept (0.5 mg/mL)

Time-point after induction	Eye	Rat n°	HRA evaluation 10 minutes after fluorescein injection (scale: 0-3 arbitrary unit)											
			Spot N°						Individual Mean	Individual SD	*Mean*	*SD*	*MEDIAN*	Interquartile range
			1	2	3	4	5	6						
Day 14	R	R#57	2	ND	ND	ND	ND	3	ND	-	***1.7***	*1.0*	***1.8***	*1.2*
		R#58	3	ND	3	3	3	3	3.0	0.0				
		R#59	1	0	0	0	0	0	0.2	0.4				
		R#60	3	1	3	2	3	3	2.5	0.8				
		R#61	ND	ND	ND	ND	ND	ND	ND	-				
		R#62	1	1	3	2	1	2	1.7	0.8				
		R#63	0	1	1	2	1	1	1.0	0.6				
		R#64	2	1	2	1	2	3	1.8	0.8				
Day 21	R	R#57	ND	ND	2	ND	ND	3	ND	-	***2.3***	*0.7*	***2.5***	*0.8*
		R#58	3	3	3	3	3	3	3.0	0.0				
		R#59	3	2	3	3	3	3	2.8	0.4				
		R#60	3	1	3	1	2	3	2.2	1.0				
		R#61	ND	ND	ND	ND	ND	ND	ND	-				
		R#62	3	2	3	3	3	3	2.8	0.4				
		R#63	0	3	1	2	3	3	2.0	1.3				
		R#64	2	1	2	1	0	1	1.2	0.8				

Table S2 A-C: Angiography scores of individual lesions (columns) for each rat (row) in the group on Day 14 and Day 21 after ChNV lesion induction. HRA scores are color-coded to produce a 'heat-map' for easy visualization. An angiography score of 0 (cyan) indicates little or no vessel leakage, whereas a score of 3 (red) indicates substantial vessel leakage. Where lesions were not evaluable for technical reasons (merging with other lesions, obscuring the field of view or where the retinal membrane was ruptured), they were categorized as 'ND'. Where more than 3 ND lesions were recorded in a given rat, the data for that rat was excluded (-) from the analysis. R# = rat number; R = right eye.
