# Supplementary material for: Successful Proof-of-Concept for Topical Delivery of Novel Peptide ALM201 with Potential Usefulness for Treating Neovascular Eye Disorders
Source: Ophthalmol Sci. 2022 Apr 4;2(2):100150. doi: 10.1016/j.xops.2022.100150 (PMC9560569; doi:10.1016/j.xops.2022.100150)
Supplement: Table S3 [file mmc6.pdf]

Treatment	Time-point after induction	Rat n°	Neovascularized lesion size (µm^3)					
			Lesion n°					
			1	*2*	*3*	*4*	*5*	*6*
**Topical vehicle (PBS)**	**Day 23**	**1**	ND	ND	156 929	198 094	310 905	ND
		**2**	95 121	149 323	380 459	298 710	138 185	102 550
		**3**	159 532	166 671	183 506	300 045	214 705	119 868
		**4**	85 736	19 683	313 213	174 708	208 283	254 880
		**5**	177 606	274 453	ND	ND	122 620	70 978
		**6**	299 817	356 897	112 469	ND	284 472	113 393
		**7**	ND	262 425	18 213	190 797	ND	127 663
		**8**	274 395	140 103	107 643	139 140	115 914	278 505
**Topical ALM201 (10 µM)**	**Day 23**	**17**	234 726	84 068	195 879	27 936	68 905	89 287
		**18**	92 063	83 413	85 970	145 405	135 118	153 173
		**19**	ND	ND	96 348	176 434	81 747	85 817
		**20**	93 738	111 163	ND	165 268	187 813	182 039
		**21**	104 790	140 012	131 688	220 781	186 520	169 027
		**22**	ND	68 451	103 295	93 973	160 369	238 435
		**23**	ND	343 944	ND	ND	48 476	107 717
		**24**	176 404	111 626	177 646	368 990	ND	ND
**Intravitreal aflibercept (0.5 mg/mL)**	**Day 23**	**57**	139 330	ND	ND	118 031	ND	104 638
		**58**	36 017	128 198	104 733	62 545	127 546	21 205
		**59**	ND	38 037	26 583	45 148	87 786	75 490
		**60**	ND	ND	43 398	24 663	13 514	44 874
		**61**	81 850	27 722	117 888	72 832	45 235	69 456
		**62**	100 383	399 458	247 481	102 288	156 242	79 505
		**63**	35 327	38 267	17 076	22 946	13 700	ND
		**64**	99 964	71 255	36 202	ND	38 607	39 524

Treatment	Time-point after induction	Rat n°	Neovascularized lesion size (µm^3)				
			Individual Mean	Individual SD	Group Mean	Group Median	Group SD
**Topical vehicle (PBS)**	**Day 23**	**R#1**	**221 976**	79718	**187 923**	**183 403**	28 575
		**R#2**	**194 058**	117459			
		**R#3**	**190 721**	61888			
		**R#4**	**176 084**	108329			
		**R#5**	**161 414**	87032			
		**R#6**	**233 410**	113244			
		**R#7**	**149 775**	103554			
		**R#8**	**175 950**	78891			
**Topical ALM201 (10 µM)**	**Day 23**	**R#17**	**116 800**	80224	**144 729**	**140 454**	33 239
		**R#18**	**115 857**	32089			
		**R#19**	**110 087**	44657			
		**R#20**	**148 004**	42846			
		**R#21**	**158 803**	41761			
		**R#22**	**132 905**	67893			
		**R#23**	**166 712**	156319			
		**R#24**	**208 667**	111241			
**Intravitreal aflibercept (0.5 mg/mL)**	**Day 23**	**R#57**	**120 666**	17495	**77 445**	**63 137**	51 245
		**R#58**	**80 041**	46672			
		**R#59**	**54 609**	25915			
		**R#60**	**31 612**	15173			
		**R#61**	**69 164**	31108			
		**R#62**	**180 893**	123087			
		**R#63**	**25 463**	10912			
		**R#64**	**57 110**	27952			

Table S3: Evaluation of ChNV lesion size on Day 23. Where lesions were not evaluable for technical reasons (merging with other lesions, obscuring the field of view or where the retinal membrane was ruptured), they were categorized as ‘ND’. Where more than 3 ND lesions were recorded in a given rat, the data for that rat was excluded (-) from the analysis. R# = rat

number. The top table lists the individual lesion volumes for each rat in each treatment group; the lower table summarizes the group statistics.
