# Supplementary material for: Successful Proof-of-Concept for Topical Delivery of Novel Peptide ALM201 with Potential Usefulness for Treating Neovascular Eye Disorders
Source: Ophthalmol Sci. 2022 Apr 4;2(2):100150. doi: 10.1016/j.xops.2022.100150 (PMC9560569; doi:10.1016/j.xops.2022.100150)
Supplement: Figure S1 [file mmc7.pdf]

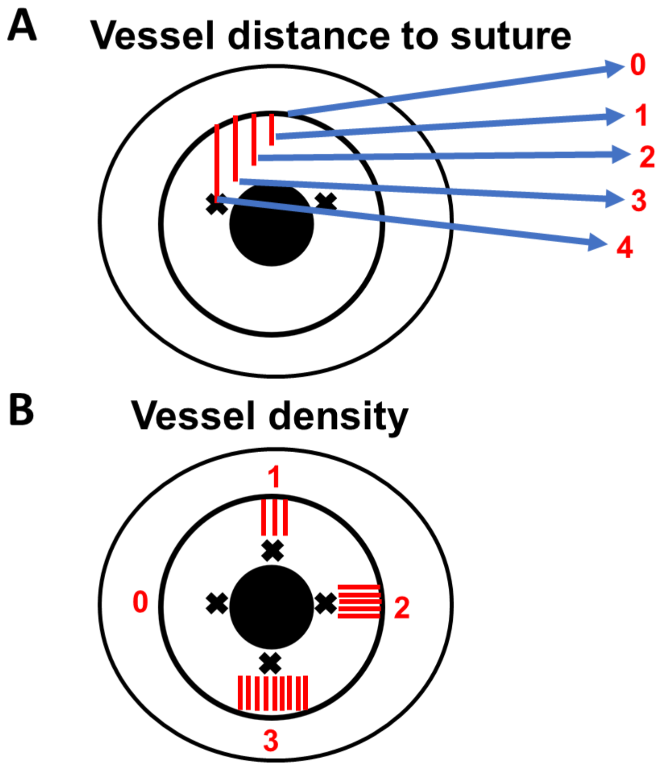

Figure S1: Clinical scoring to quantitate neovascular response in the corneal suture model of neovascularization in rat eyes. **(A)** Vessel distance to suture scored on a scale of 0-4 where increasing numbers indicate increasing vascular distance. **(B)** Vessel density scored on a scale of 0-3 where increasing numbers indicate increasing vessel density.
