# Supplementary material for: Successful Proof-of-Concept for Topical Delivery of Novel Peptide ALM201 with Potential Usefulness for Treating Neovascular Eye Disorders
Source: Ophthalmol Sci. 2022 Apr 4;2(2):100150. doi: 10.1016/j.xops.2022.100150 (PMC9560569; doi:10.1016/j.xops.2022.100150)
Supplement: Figure S2 [file mmc8.pdf]

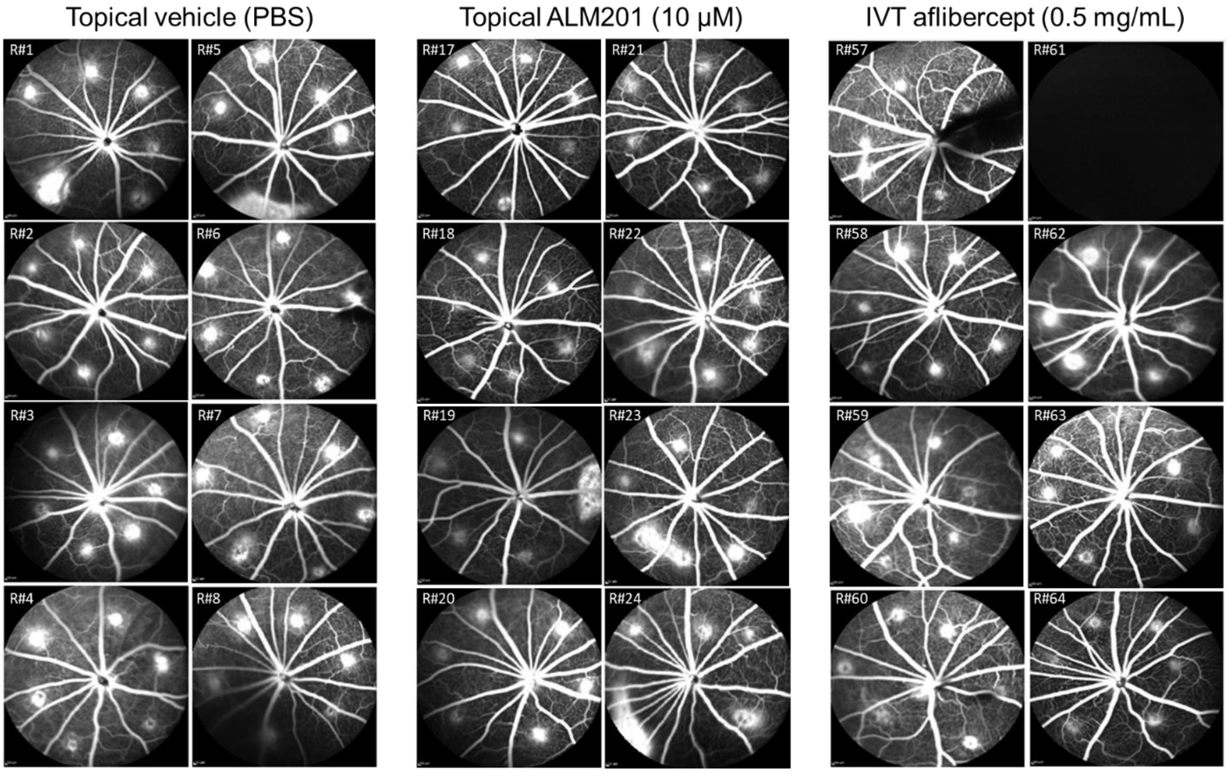

Figure S2: Images of fluorescein angiography from the right eye of each rat in each treatment group on Day 21, 10min after fluorescein injection. A clear image was not obtained for rat #61 in the intravitreal aflibercept group. R# = rat number.
