# Supplementary material for: Successful Proof-of-Concept for Topical Delivery of Novel Peptide ALM201 with Potential Usefulness for Treating Neovascular Eye Disorders
Source: Ophthalmol Sci. 2022 Apr 4;2(2):100150. doi: 10.1016/j.xops.2022.100150 (PMC9560569; doi:10.1016/j.xops.2022.100150)
Supplement: Figure S3 [file mmc9.pdf]

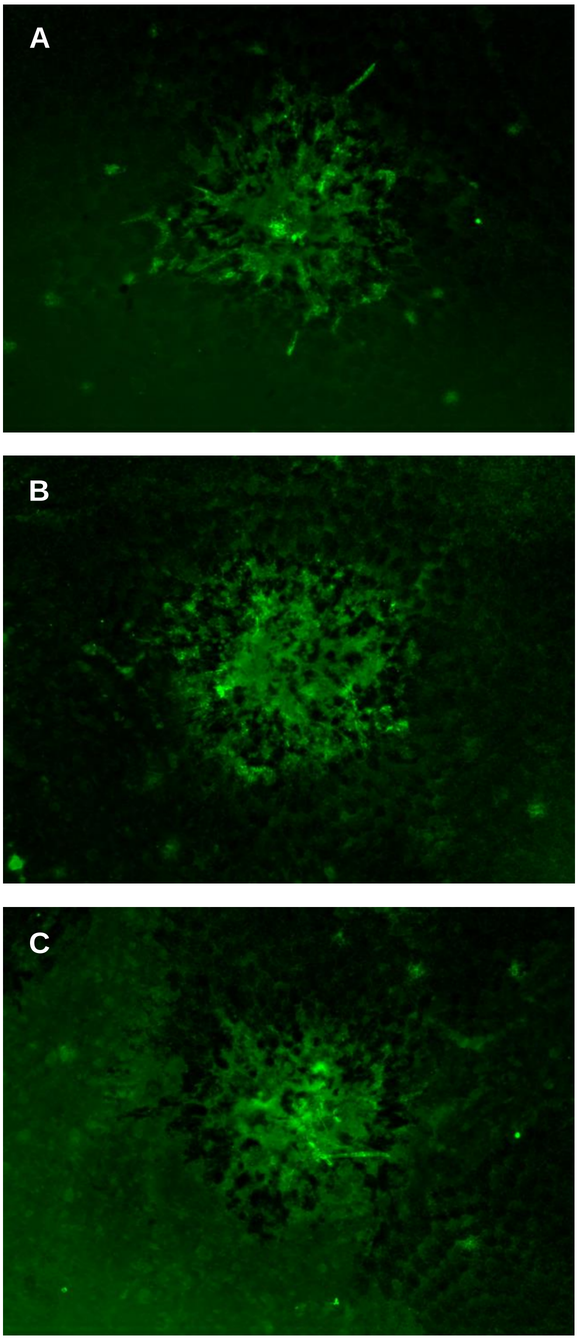

Figure S3: Flat-mount images of representative laser-induced choroidal lesions in the eyes of rats treated with either (A) Topical vehicle (PBS), (B) Topical ALM201 (10µM), or (C) Intravitreal aflibercept (5mg/mL; Eylea®) stained with FITC-Isolectin B4 at study termination (Day 23). These images show a 2-dimensional image whereas calculations of lesion volume were based on z-stack images of the vessel borders (see *Methods* for details).
