# Supplementary material for: Successful Proof-of-Concept for Topical Delivery of Novel Peptide ALM201 with Potential Usefulness for Treating Neovascular Eye Disorders
Source: Ophthalmol Sci. 2022 Apr 4;2(2):100150. doi: 10.1016/j.xops.2022.100150 (PMC9560569; doi:10.1016/j.xops.2022.100150)
Supplement: Appendix [file mmc10.pdf]

Appendix 1: Scales for the ocular examinations

Scale from McDonald and Shadduck

- **CONJUNCTIVAE**

a. Conjunctival Congestion

- Normal. May appear blanched to reddish pink without perilimbal injection (except at 12:00 and 6:00 o'clock positions) with vessels of the palpebral and bulbar conjunctiva easily observed **0**
- A flushed, reddish color predominately confined to the palpebral conjunctiva with some perilimbal injection but primarily confined to the lower and upper parts of the eye from the 4:00 to 7:00 and 11:00 to 1:00 o'clock positions **1**
- Bright red color of the palpebral conjunctiva with accompanying perilimbal injection covering at least 75% of the circumference of the perilimbal region **2**
- Dark, beefy red color with congestion of both the bulbar and the palpebral conjunctiva along with pronounced perilimbal injection and the presence of petechia on the conjunctiva. The petechia generally predominate along the nictitating membrane and the upper palpebral conjunctiva **3**

b. Conjunctival Swelling

- Normal or no swelling of the conjunctival tissue **0**
- Swelling above normal without eversion of the lids (can be easily ascertained by noting that the upper and lower eyelids are positioned as in the normal eye); swelling generally starts in the lower cul-de-sac near the inner canthus which needs slit-lamp examination **1**
- Swelling with misalignment of the normal approximation of the upper and lower eyelids; primarily confined to the upper eyelid so that in the initial stages the misapproximation of the eyelids begins by partial eversion of the upper eyelid. In this stage, swelling is confined generally to the upper eyelid, although it exists in the lower cul-de-sac (observed best with the slit-lamp) **2**
- Swelling definite with partial eversion of the upper and lower eyelids essentially equivalent. This can be easily ascertained by looking at the animal head-on and noticing the positioning of the eyelids; if the eyes margins do not meet, eversion has occurred **3**
- Eversion of the upper eyelid is pronounced with less pronounced eversion of the lower eyelid. It is difficult to retract the lids and observe the perilimbal region **4**

c. Conjunctival Discharge

Discharge is defined as a whitish gray precipitate, which should not be confused with the small amount of clear, inspissated, mucoid material that can be formed in the medial canthus of a substantial number of rabbit eyes. This material can be removed with cotton swab before the animals are used.

- Normal. No discharge **0**
- Discharge above normal and present on the inner portion of the eye but not on the lids or hairs of the eyelids. One can ignore the small amount that is in the inner and outer canthus if it has not been removed prior to starting the study **1**
- Discharge is abundant, easily observed, and has collected on the lids and around the hairs of the eyelids **2**
- Discharge has been flowing over the eyelids so as to wet the hairs substantially on the skin around the eye **3**

♦ AQUEOUS FLARE

The intensity of the Tyndall phenomenon is scored by comparing the normal Tyndall effect observed when the slit-lamp beam passes through the lens with that seen in the anterior chamber. The presence of aqueous flare is presumptive evidence of breakdown of the blood-aqueous barrier.

- Absence of visible light beam light in the anterior chamber (no Tyndall effect) **0**
- The Tyndall effect is barely discernible. The intensity of the light beam in the anterior chamber is less than the intensity of the slit beam as it passes through the lens **1**
- The Tyndall beam in the anterior chamber is easily discernible and is equal in intensity to the slit beam as it passes through the lens **2**
- The Tyndall beam in the anterior chamber is easily discernible; its intensity is greater than the intensity of the slit beam as it passes through the lens **3**

♦ IRIS

In the following definitions the primary, secondary and tertiary vessels are utilized as an aid to determining a subjective ocular score for iris involvement. The assumption is made that the greater the hyperemia of the vessels and the more the secondary and tertiary vessels are involved, the greater the intensity of iris involvement. The scores range from 0 to +4.

- Normal iris without any hyperemia of the iris vessels. Occasionally around the 12:00 to 1:00 o'clock position near the pupillary border and the 6:00 and 7:00 o'clock position near the pupillary border there is a small area about 1-3 mm in diameter in which both the secondary and tertiary vessels are slightly hyperemic **0**
- Minimal injection of secondary vessels but not tertiary. Generally, it is uniform, but may be of greater intensity at the 1:00 or 6:00 o'clock position. If it is confined to the 1:00 or 6:00 o'clock position, the tertiary vessels must be substantially hyperemic **1**
- Minimal injection of tertiary vessels and minimal to moderate injection of the secondary vessels **2**
- Moderate injection of the secondary and tertiary vessels with slight swelling of the iris stroma (this gives the iris surface a slightly rugose appearance, which is usually most prominent near the 3:00 and 9:00 o'clock positions) **3**
- Marked injection of the secondary and tertiary vessels with marked swelling of the iris stroma. The iris appears rugose; may be accompanied by hemorrhage (hyphema) in the anterior chamber **4**

♦ CORNEA

a. Opacities

The scoring scheme measures the severity of corneal cloudiness and the area of the cornea involved. Severity of corneal cloudiness is graded as follows:

- Normal cornea. Appears with the slit lamp as having a bright gray line on the epithelial surface and a bright gray line on the endothelial surface with a marblelike gray appearance of the stroma **0**
- Some loss of transparency. Only the anterior half of the stroma is involved as observed with an optical section of the slit lamp. The underlying structures are clearly visible with diffuse illumination, although some cloudiness can be readily apparent with diffuse illumination **1**
- Moderate loss of transparency. In addition to involving the anterior stroma, the cloudiness extends all the way to the endothelium. The stroma has lost its marblelike appearance and is homogeneously white. With diffuse illumination, underlying structures are clearly visible **2**
- Involvement of the entire thickness of the stroma. With optical section, the endothelial surface is still visible. However, with diffuse illumination the underlying structures are just barely visible (to the extent that the observer is still able to grade flare, iritis, observe for pupillary response, and note lenticular changes) **3**

- Involvement of the entire thickness of the stroma. With optical section, cannot clearly visualize the endothelium. With diffuse illumination, the underlying structures cannot be seen. Cloudiness removes the capability for judging and grading aqueous flare, iritis, lenticular changes and pupillary response **4**

b. % Area of Corneal Opacity

- -Normal cornea with no area of cloudiness **0**
- -1-25% area of stromal cloudiness **1**
- -26-50% area of stromal cloudiness **2**
- -51-75% area of stromal cloudiness **3**
- -76-100% area of stromal cloudiness **4**

c. Corneal Vascularization

Pannus is vascularization or the penetration of new blood vessels into the corneal stroma. The vessels are derived from the limbal vascular loops.

- No corneal vascularization (pannus) **0**
- Vascularization is present but vessels have not invaded the entire corneal circumference. Where localized vessel invasion has occurred, they have not penetrated beyond 2 mm **1**
- Vessels have invaded 2 mm or more around the entire corneal circumference **2**

d. Corneal Staining

- Absence of fluorescein staining **0**
- Slight fluorescein staining confined to a small focus. With diffuse illumination the underlying structures are easily visible. The outline of the pupillary margin is as if there were no fluorescein staining **1**
- Moderate fluorescein staining confined to a small focus. With diffuse illumination the underlying structures are clearly visible, although there is some loss of detail **2**
- Marked fluorescein staining. Staining may involve a larger portion of the cornea. With diffuse illumination underlying structures are barely visible, but are not completely obliterated **3**
- Extreme fluorescein staining. With diffuse illumination the underlying structures cannot be observed **4**

Note: KIKKAWA reported that 10-20% of rabbits examined exhibited focal, punctuate fluorescein staining normally. There may be involvement of the whole cornea, or the focal may be limited to one area. (KIKKAWA Y. Normal corneal staining with fluorescein. **Exp. Eye Res.** 1972; **14**: 13-20).

e. % Area of Corneal Staining

- No cornea of fluorescein staining **0**
- 1-25% area of fluorescein staining **1**
- 26-50% area of fluorescein staining **2**
- 51-75% area of fluorescein staining **3**
- 76-100% area of fluorescein staining **4**

Note: The entire area of the cornea that contains stain is scored, regardless of the varying intensities that may be present.

♦ LENS

The lens should be evaluated routinely during ocular evaluations and graded as either 0 (normal) or 1 (abnormal). The presence of lenticular opacities should be described.

♦ FUNDUSCOPY

The fundus should be evaluated routinely during ocular evaluations and graded as either 0 (normal) or 1 (abnormal). Whenever a fundus or a vitreous body is scored “1”, a text field will be used to describe the lesion, its location and its grade.
